# Targeting eIF4A triggers an interferon response to synergize with chemotherapy and suppress triple-negative breast cancer

**DOI:** 10.1172/JCI172503

**Published:** 2023-12-15

**Authors:** Na Zhao, Elena B. Kabotyanski, Alexander B. Saltzman, Anna Malovannaya, Xueying Yuan, Lucas C. Reineke, Nadia Lieu, Yang Gao, Diego A. Pedroza, Sebastian J. Calderon, Alex J. Smith, Clark Hamor, Kazem Safari, Sara Savage, Bing Zhang, Jianling Zhou, Luisa M. Solis, Susan G. Hilsenbeck, Cheng Fan, Charles M. Perou, Jeffrey M. Rosen

**Affiliations:** 1 Department of Molecular and Cellular Biology,; 2 Mass Spectrometry Proteomics Core,; 3 Department of Biochemistry and Molecular Pharmacology, and; 4 Department of Neuroscience, Baylor College of Medicine, Houston, Texas, USA.; 5 Texas A&M Health Science Center, Houston, Texas, USA.; 6 Lester and Sue Smith Breast Center, Baylor College of Medicine, Houston, Texas, USA.; 7 Department of Translational Molecular Pathology, The University of Texas MD Anderson Cancer Center, Houston, Texas, USA.; 8 Lineberger Comprehensive Cancer Center, University of North Carolina, Chapel Hill, North Carolina, USA.

**Keywords:** Oncology, Breast cancer, Immunotherapy, Translation

## Abstract

Protein synthesis is frequently dysregulated in cancer and selective inhibition of mRNA translation represents an attractive cancer therapy. Here, we show that therapeutically targeting the RNA helicase eIF4A with zotatifin, the first-in-class eIF4A inhibitor, exerts pleiotropic effects on both tumor cells and the tumor immune microenvironment in a diverse cohort of syngeneic triple-negative breast cancer (TNBC) mouse models. Zotatifin not only suppresses tumor cell proliferation but also directly repolarizes macrophages toward an M1-like phenotype and inhibits neutrophil infiltration, which sensitizes tumors to immune checkpoint blockade. Mechanistic studies revealed that zotatifin reprograms the tumor translational landscape, inhibits the translation of *Sox*4 and *Fgfr1*, and induces an interferon (IFN) response uniformly across models. The induction of an IFN response is partially due to the inhibition of *Sox4* translation by zotatifin. A similar induction of IFN-stimulated genes was observed in breast cancer patient biopsies following zotatifin treatment. Surprisingly, zotatifin significantly synergizes with carboplatin to trigger DNA damage and an even heightened IFN response, resulting in T cell–dependent tumor suppression. These studies identified a vulnerability of eIF4A in TNBC, potential pharmacodynamic biomarkers for zotatifin, and provide a rationale for new combination regimens consisting of zotatifin and chemotherapy or immunotherapy as treatments for TNBC.

## Graphical abstract

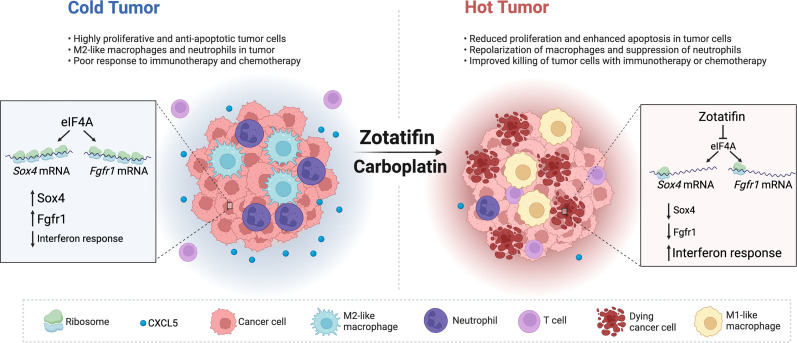

## Introduction

Triple-negative breast cancer (TNBC) is a heterogeneous group of breast cancers defined by the absence of estrogen receptor (ER), progesterone receptor (PR), or human epidermal growth factor receptor 2 (HER2), and is minimally classified into 4 genomic subtypes (1). TNBC patients have pathological complete response (pCR) rates of 30% to 53% when treated with a neoadjuvant anthracycline/taxane–containing regimen (2), and recently in a subset of patients pCR rates were improved following treatment with immune checkpoint blockade (ICB) (3). Recent improvements have also been made in treating metastatic TNBC with an antibody-drug conjugate (4) or ICB in combination with chemotherapy in PD-L1^+^ TNBC (5, 6). However, there is a critical need to identify therapeutic vulnerabilities and treatments that can potentiate the response to chemotherapy and immunotherapy.

One key target for cancer therapy is protein synthesis, which is frequently dysregulated in cancer. Nearly all critical oncogenic signaling pathways ultimately rewire the translational machinery to support tumorigenesis (7). Of all steps in protein synthesis, translation initiation is the rate-limiting step and is subject to extensive regulation (8). Oncogenic signaling pathways promote translation initiation mainly through stimulation of the eukaryotic translation initiation factor 4F (eIF4F) complex. The eIF4F complex consists of 3 components, of which eIF4A is an RNA helicase that catalyzes the unwinding of secondary structure in the 5′-untranslated region (5′-UTR) of mRNAs to facilitate translation initiation (9). Multiple tumor-promoting genes that contain structured 5′-UTRs require enhanced eIF4A activity for translation (10–13). The natural compound rocaglamide A (RocA) and its derivatives (rocaglates) can target the bimolecular cavity formed by eIF4A and polypurine RNA (14). This interaction with rocaglate clamps eIF4A on mRNAs that contain polypurine motifs in their 5′-UTR and results in sequence-selective translation repression and translatome remodeling by blocking scanning of the preinitiation complex and other mechanisms (15–17). This is in contrast with hippuristanol, an initiation inhibitor that binds to the C-terminus of eIF4A and blocks RNA binding in a sequence-independent manner (18, 19). Several studies have reported a dependency on eIF4A in different cancers, suggesting the therapeutic potential of rocaglates (12, 13, 20–22). However, these natural compounds do not possess good drug properties and their effects on the tumor immune microenvironment are not well defined.

Zotatifin (eFT226) is a chemically designed rocaglate derivative with improved drug-like properties and is the first-in-class eIF4A inhibitor (23). Zotatifin is currently undergoing a phase I/II clinical trial in KRAS-mutant tumors and ER^+^ breast cancers (ClinicalTrials.gov NCT04092673). However, patients with TNBC are not enrolled in the trial due to a lack of preclinical studies, and pharmacodynamic biomarkers for zotatifin remain to be identified. Zotatifin has been shown previously to inhibit tumor growth in several immunocompromised mouse models (20). However, since the immune system plays a crucial role in both tumor development and treatment response, it is crucial to examine zotatifin in immunocompetent preclinical models. Moreover, studies on the interaction of zotatifin with chemotherapy are important because chemotherapy remains the primary systemic treatment option for patients with TNBC and many other solid cancers. We previously developed multiple novel syngeneic TNBC genetically engineered mouse (GEM) models across different intrinsic molecular subtypes, which have been characterized both genetically and with respect to their immune microenvironments (24–30). Here, we tested the therapeutic efficacy of zotatifin both as a monotherapy and in combination with immunotherapy or chemotherapy in these GEM models. We found that zotatifin exerts pleiotropic effects on both tumor cells and the immune microenvironment and synergizes with carboplatin in mounting an interferon (IFN) response, resulting in a T cell–dependent suppression of tumor growth.

## Results

### Zotatifin monotherapy inhibits tumor growth in a cohort of syngeneic Trp53-null mammary tumor models.

**Figure 1:**
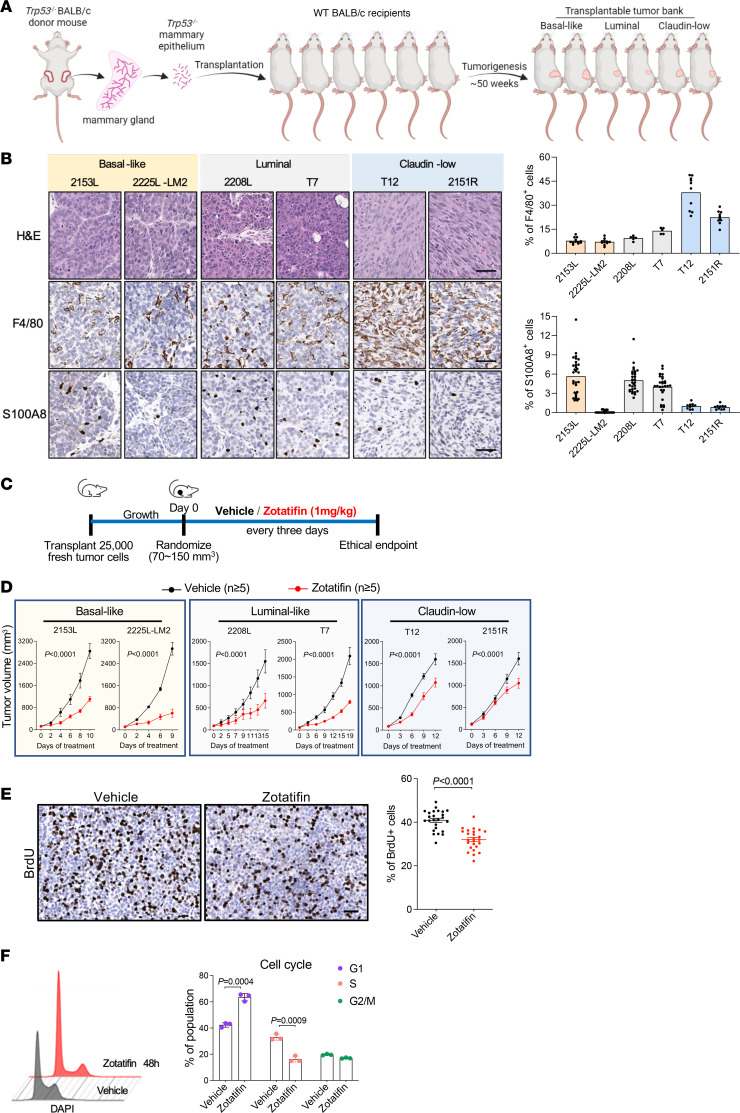
Zotatifin monotherapy inhibits tumor growth in a cohort of *Trp53*-null preclinical models. (**A**) Scheme depicting the generation of syngeneic *Trp53*-null preclinical models. Donor mammary epithelium from *Trp53*-null BALB/c mice was transplanted in situ into cleared mammary fat pads of wild-type recipient hosts, resulting in the derivation of heterogeneous, *Trp53*-null, transplantable mammary tumors representative of the different intrinsic molecular subtypes of human breast cancer. (**B**) Top: Representative images of H&E staining and IHC staining of F4/80 and S100A8 in *Trp53*-null models used in this study. Scale bars: 50 μm. Bottom: Quantification of IHC staining. Three to 6 representative ×20 images were analyzed for each tumor. (**C**) Schematic of animal treatment. Freshly dissociated tumor cells were injected into the fourth mammary fat pad of BALB/c mice. Mice were randomized and treatment was initiated when tumors reach a volume of 70–150 mm^3^. Mice were treated with either vehicle or zotatifin every 3 days until ethical endpoint. (**D**) Tumor growth curves of BALB/c mice treated with either vehicle or zotatifin. *n =* 6 for 2225L-LM2 and *n =* 5 for all other models in each treatment arm. Data are presented as mean ± SEM and were analyzed using 2-way ANOVA with Bonferroni’s multiple-comparison test. (**E**) Left: Representative images of IHC staining of BrdU in ethical endpoint 2153L tumor tissues. Scale bar: 50 μm. Right: Quantification of IHC staining. Five representative ×20 images were analyzed for each tumor. *n =* 5 biological replicates. Data are presented as mean ± SEM and were analyzed using 2-tailed, unpaired Student’s *t* test. (**F**) Left: Representative images of cell cycle distribution of 2153L cells that were cultured in complete medium and treated with vehicle or 40 nM zotatifin for 48 hours. Right: Quantification of the cell cycle phases from 3 independent experiments. Data are presented as mean ± SD and were analyzed using 2-tailed, unpaired Student’s *t* test.

As *TP53* is the most frequently altered gene in TNBC (31), syngeneic TNBC GEM models were generated previously by in situ transplantation of donor mammary epithelium from *Trp53*-null BALB/c mice into wild-type recipient hosts (Figure 1A). This resulted in the derivation of heterogeneous *Trp53*-null transplantable mammary tumors. Detailed genomic characterization has revealed that these tumors are representative of the different intrinsic molecular subtypes of human breast cancer, including the basal-like, luminal-like, and claudin-low subtypes (24–27). Besides the apparent differences in tumor histology, these tumor models also exhibited variable infiltration of myeloid cells, including macrophages and neutrophils, as illustrated by the immunostaining of F4/80 (macrophage marker) and S100A8 (neutrophil marker) (32), respectively (Figure 1B). Tumors of the claudin-low subtype (T12 and 2151R) were more mesenchymal and highly infiltrated with macrophages (Figure 1B). This correlation of mesenchymal features with macrophage infiltration has also been observed in human TNBC (33). We first determined the effect of zotatifin monotherapy in 6 GEM models across 3 subtypes. To minimize intertumoral variation between mice, we transplanted freshly dissociated tumor cells instead of tumor chunks into the mammary fat pad of BALB/c mice. When tumors reached approximately 100 mm^3^ in volume, we randomized the mice and started treatment with either vehicle or zotatifin at 1 mg/kg every 3 days (Figure 1C). Most of these models are so aggressive that they rapidly reach the ethical endpoint within 1 to 2 weeks after randomization, but zotatifin treatment slowed tumor growth without obvious body weight loss except for those caused by tumor weight reduction in 2225L-LM2 (Figure 1D and Supplemental Figure 1, A and B; supplemental material available online with this article; https://doi.org/10.1172/JCI172503DS1). Interestingly, in 2151R, vehicle-treated mice started to lose body weight starting on day 9, possibly due to cachexia induced by enlarged tumors, but mice in the zotatifin treatment group were not affected (Supplemental Figure 1B). Besides GEM models, we also tested 4T1 and E0771, two TNBC cell line models. Whereas zotatifin treatment reduced the 4T1 tumor volume, E0771 was completely resistant (Supplemental Figure 1C). Neither model showed body weight loss (Supplemental Figure 1D). These data suggest that zotatifin is an effective and well-tolerated therapy for the majority of TNBC models.

Next, we examined the effect of zotatifin on cell proliferation by measuring the level of BrdU incorporation in tumors. Zotatifin treatment suppressed DNA synthesis in 2153L, 2225L-LM2, and 2208L tumors in vivo (Figure 1E and Supplemental Figure 1, E and F). To further study the inhibition of cell proliferation, we treated 2153L primary tumor cells in vitro and found that zotatifin suppressed the G_1_/S cell cycle transition (Figure 1F). These data indicate that zotatifin can inhibit proliferation in a tumor cell–autonomous manner.

### Zotatifin suppresses the infiltration of neutrophils and M2-like macrophages and sensitizes tumors to ICB.

**Figure 2:**
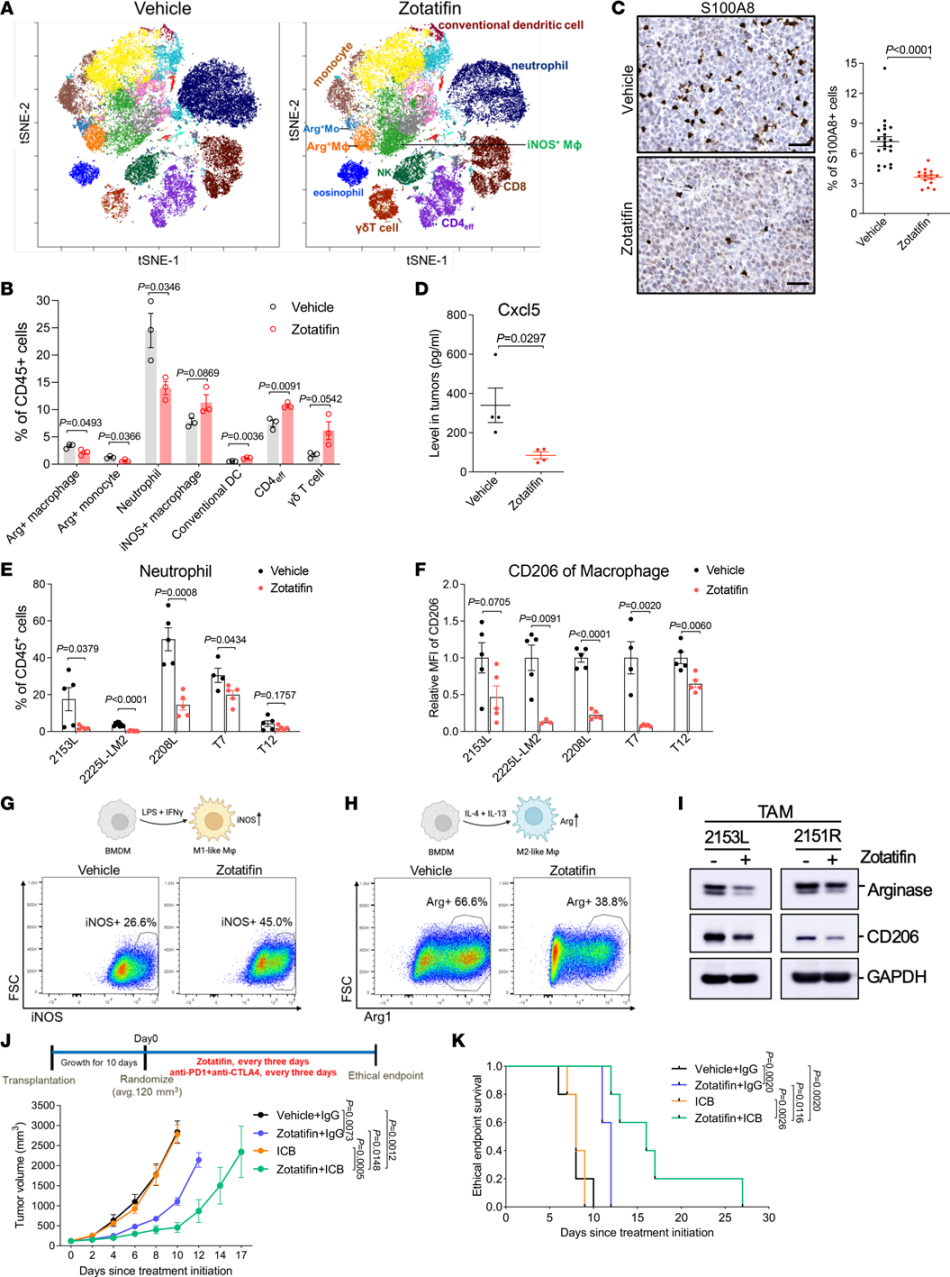
Zotatifin monotherapy alters the tumor immune microenvironment and sensitizes tumors to immune checkpoint blockade. (**A**) t-Distributed stochastic neighbor embedding (t-SNE) projection of tumor-infiltrating immune cells in 2153L tumors that were treated for 7 days and analyzed using mass cytometry. Data from 3 biological replicates of each group were concatenated before t-SNE and FlowSOM analysis. Equal cell numbers are shown for each group. (**B**) Quantification of major immune cell populations in mass cytometry analysis. *n =* 3 per group. (**C**) Left: Representative images of IHC staining of S100A8 in 2153L tumors treated with vehicle or zotatifin. Scale bars: 50 μm. Right: Quantification of staining. Multiple ×20 images were analyzed for each tumor. *n =* 5 biological replicates per group. (**D**) Luminex array detection of Cxcl5 levels in 2153L tumor lysates. *n =* 4 biological replicates per group. (**E** and **F**) Flow cytometry analysis of tumor-infiltrating neutrophils (**E**) and CD206 median fluorescence intensity (MFI) in tumor-infiltrating macrophages (**F**) in ethical endpoint tumors. *n* ≥ 4 per group in all models. In **B**–**F**, data are presented as mean ± SEM and were analyzed using 2-tailed, unpaired Student’s *t* test. (**G**) Flow cytometry analysis of iNOS expression in BMDMs that were treated with vehicle or zotatifin in the presence of LPS and IFN-γ. (**H**) Flow cytometry analysis of Arg1 expression in BMDMs that were treated with vehicle or zotatifin in the presence of IL-4 and IL-13. (**I**) Immunoblotting of TAMs that were separated from untreated tumors and treated with vehicle or zotatifin for 24 hours in vitro. See complete unedited blots in the supplemental material. (**J**) Growth curves of 2153L tumors treated with indicated drugs. *n =* 5 per group. Data are presented as mean ± SEM and were analyzed using 2-way ANOVA with Tukey’s multiple-comparison test. (**K**) Kaplan-Meier survival curves of 2153L tumor–bearing mice from treatment start time. The log-rank test was used to test for the significant differences of curves. *n =* 5 per group.

In addition to tumor cell–autonomous effects, we interrogated the tumor immune microenvironment in these syngeneic GEM models. To determine whether zotatifin affects tumor-infiltrating immune cells, we carried out mass cytometry for 2153L tumors that were treated with vehicle or zotatifin for 7 days in vivo. Zotatifin inhibited the infiltration of the following populations: immunosuppressive M2-like macrophages (CD11b^+^F4/80^+^Arg1^+^PD-L1^+^), arginase 1^+^ (Arg1^+^) monocytes that are commonly considered as monocytic myeloid-derived suppressor cells (MDSCs), and neutrophils (CD11b^+^Ly6C^lo^Ly6G^+^), which are commonly defined as granulocytic MDSCs (Figure 2, A and B). The reduction in neutrophil infiltration by zotatifin was also confirmed by immunostaining for the neutrophil marker S100A8 in 2153L, 2225L-LM2, and 2208L tumor tissues (Figure 2C and Supplemental Figure 2, A and B). Concordantly, we also observed decreased production of Cxcl5 (Figure 2D), the chemokine that stimulates the chemotaxis of neutrophils possessing angiogenic properties (34). On the other hand, zotatifin promoted the infiltration of the following populations: proinflammatory M1-like macrophages (CD11b^+^F4/80^+^Arg1^–^iNOS^+^MHCII^+^), conventional dendritic cells (CD11b^–^CD11c^+^CD103^+^MHCII^+^), effector CD4^+^ T cells (CD4^+^FoxP3^–^), and γδ T cells (CD3^+^ TCRβ^–^CD4^–^CD8^–^TCRδ^+^) (Figure 2, A and B). Decreased neutrophil infiltration and reduced expression of the immunosuppressive macrophage marker CD206 upon zotatifin treatment were consistently observed across multiple mouse models, as analyzed by flow cytometry (Figure 2, E and F), indicating a general effect of zotatifin on the tumor immune microenvironment.

We next explored whether zotatifin can directly repolarize macrophages. When bone marrow–derived macrophages (BMDMs) were cultured in the presence of LPS and IFN-γ, the stimuli that induce macrophage polarization toward an M1-like tumor-inhibitory phenotype (35), zotatifin treatment increased the percentage of iNOS^+^ cells as compared with the vehicle (Figure 2G). On the other hand, in the presence of IL-4 and IL-13, which are immunosuppressive macrophage polarization stimuli (35), zotatifin reduced the percentage of Arg1^+^ BMDMs (Figure 2H). Additionally, we isolated macrophages from untreated 2153L and 2151R tumors and found that zotatifin treatment suppressed the expression of Arg1 and CD206 in these tumor-associated macrophages (TAMs) (Figure 2I). These findings suggest that zotatifin directly promotes the polarization of macrophages toward a tumor-inhibitory phenotype and suppresses their differentiation toward an immunosuppressive phenotype. To confirm the importance of macrophages in the response to zotatifin monotherapy, we depleted TAMs using an anti-Csf1r antibody (Supplemental Figure 2C) (30). We observed that depleting macrophages partially abolished the response to zotatifin, suggesting that the repolarization of macrophages contributed to the zotatifin-monotherapy response (Supplemental Figure 2D).

High infiltration of immunosuppressive macrophages and neutrophils is associated with a poor response to ICB (28, 36–39). Given that zotatifin reduced these immunosuppressive myeloid populations, we hypothesized that it could sensitize tumors to ICB. We observed that 2153L tumors were completely resistant to CTLA-4 and PD-1 blockade, but the combination with zotatifin sensitized this immune-cold tumor model to ICBs and enhanced survival compared with monotherapy (Figure 2, J and K). These data demonstrate that in addition to its tumor cell–autonomous effects, zotatifin also reprogrammed the tumor immune microenvironment to facilitate the therapeutic benefit of checkpoint inhibition in immune-cold TNBC.

### Zotatifin remodels the proteomic landscape and inhibits the translation of Sox4 and Fgfr1.

**Figure 3:**
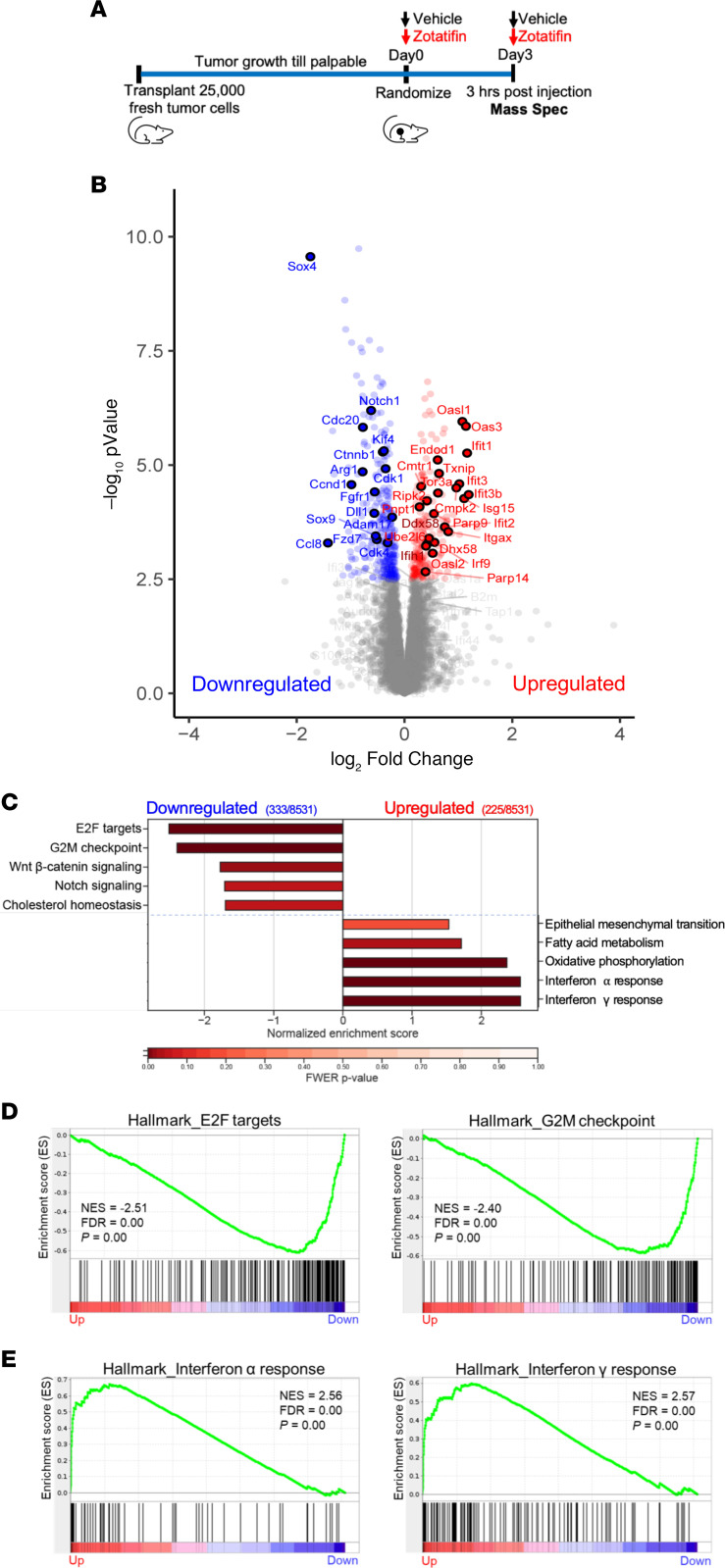
Zotatifin remodels the proteomic landscape of 2153L tumors. (**A**) Scheme of sample collection strategy for mass spectrometry. Freshly dissociated 2153L tumor cells were transplanted into the fourth mammary fat pad of BALB/c mice and were allowed to grow until palpable. Mice then were randomized and treated with either vehicle or zotatifin for 2 doses spanning 3 days. Tumor tissues were collected 3 hours after the second injection. (**B**) Volcano plot showing relative fold change (log_2_) in protein abundance versus −log_10_(*P* values) from 2153L tumors treated with zotatifin compared with vehicle. Proteins that demonstrate a significant change in expression (FDR *q* < 0.05) are colored, with decreased expression on the left in blue and increased expression on the right in red. Genes that are critically involved in cell proliferation, stem cell signaling, and IFN response pathways are labeled. *n =* 4 biological replicates per arm. Statistical significance was determined by 2-tailed, unpaired moderated *t* test. (**C**) GSEA of mass spectrometry results was performed with the MSigDB hallmarks data set and is summarized as the normalized enrichment score (NES) in vehicle- or zotatifin-treated 2153L tumor tissues. Top pathways that have a family-wise error rate (FWER) *P* < 0.25 are displayed. FWER *P* values for each pathway are denoted by color. (**D**) GSEA enrichment plots for Hallmark E2F targets and G_2_M checkpoint signatures that are downregulated in zotatifin-treated tumors compared with vehicle. (**E**) GSEA enrichment plots for Hallmark IFN-α response and IFN-γ response signatures that are upregulated in zotatifin-treated tumors compared with vehicle.

Next, we explored the mechanism underlying the therapeutic effects of zotatifin monotherapy. Since eIF4A, the major zotatifin target, primarily affects protein synthesis, we applied quantitative proteomics to unbiasedly investigate changes in steady-state protein levels caused by acute zotatifin treatment in one of the responsive tumors in vivo. 2153L tumors were treated with either vehicle or zotatifin for 2 doses spanning 3 days before analysis by tandem mass tag mass spectrometry (TMT-MS) (Figure 3A). This treatment design allows for the interrogation of both short-lived and long-lived direct protein targets of zotatifin, although secondary effects might also be captured. This analysis identified significant alterations in the abundance of 558 of 8531 detected proteins upon zotatifin treatment that reached the criteria of FDR less than 0.05, with 333 proteins showing decreased expression and 225 proteins showing increased expression (Figure 3B and Supplemental Table 1). Hallmark gene set enrichment analysis (GSEA) revealed that zotatifin downregulated the expression of proteins involved in cell cycle progression and stem cell signaling pathways, including E2F targets, G_2_/M checkpoint, Wnt/β-catenin, and Notch signaling (Figure 3, B–D, and Supplemental Table 2). On the other hand, proteins involved in IFN-α and IFN-γ responses were induced in response to zotatifin treatment (Figure 3, B, C, and E, and Supplemental Table 3).

**Figure 4:**
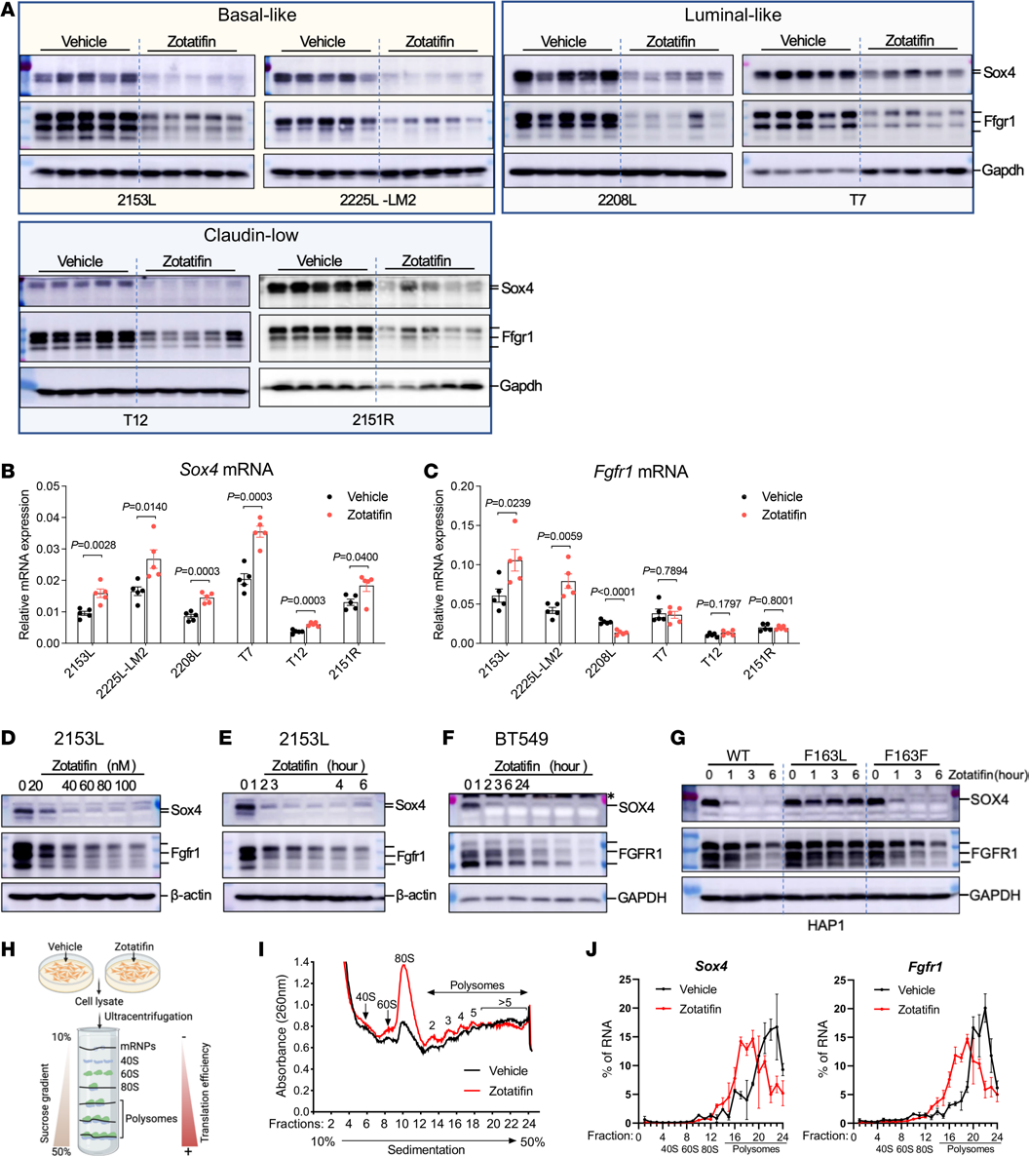
Zotatifin inhibits the translation of *Sox4* and *Fgfr1* mRNAs. (**A**) Immunoblotting analysis of tumors that were treated with vehicle or zotatifin in vivo. *n* = 5 biological replicates per group. (**B** and **C**) qPCR analysis for *Sox4* (**B**) and *Fgfr1* (**C**) mRNA expression in tumors that were treated with vehicle or zotatifin in vivo. Data are presented as mean ± SEM and were analyzed using 2-tailed, unpaired Student’s *t* test. *n =* 5 biological replicates per group. (**D**) Immunoblotting analysis of 2153L cells that were treated with different concentrations of zotatifin for 6 hours in vitro. (**E**) Immunoblotting analysis of 2153L cells that were treated with 40 nM zotatifin for different time periods. (**F**) Immunoblotting analysis of BT549 cells that were treated with 40 nM zotatifin for different time periods. *Denotes a nonspecific band. In **D**–**F**, data are representative of 3 independent experiments. (**G**) Immunoblotting analysis of HAP1 cells that were treated with 40 nM zotatifin in vitro. Data are representative of 2 independent experiments. (**H**) Illustration for polysome profiling analysis. (**I** and **J**) Polysome profiling of 2153L cells that were treated with vehicle or 40 nM zotatifin for 2 hours. (**I**) Representative polysome profiles from 3 biological replicates. (**J**) Distribution of *Sox4* and *Fgfr1* mRNAs across the different fractions. Data are presented as mean ± SEM of 3 biological replicates. See complete unedited blots in the supplemental material.

We next validated several targets from the proteomic analysis. Sox4 and Fgfr1 were chosen because Sox4 is the most significantly downregulated protein and Fgfr1 is an important receptor tyrosine kinase. First, we performed immunoblotting on a biological replicate of 2153L tumor samples and observed a dramatic reduction in Sox4 and Fgfr1 protein expression in the zotatifin treatment group (Figure 4A). To determine whether this is a general phenomenon, we performed immunoblotting on the other 5 GEM models. Strikingly, zotatifin treatment caused a uniform and potent downregulation of Sox4 and Fgfr1 protein expression in all the GEM models, despite intertumoral variation (Figure 4A). This was also confirmed in 4T1 tumors (Supplemental Figure 3A). Interestingly, Sox4 was not detectable by immunoblotting in the zotatifin-resistant E0771, although zotatifin was able to downregulate Fgfr1 expression in this cell line (Supplemental Figure 3B). To investigate at which step this regulation occurs, we quantified *Sox4* and *Fgfr1* RNA expression and found no reduction following zotatifin treatment in any of the GEM models, except for *Fgfr1* in 2208L (Figure 4, B and C), indicating posttranscriptional regulation. Next, we conducted a dose-response analysis of zotatifin treatment in vitro using primary cells derived from 2153L tumors. We again observed a dose-dependent reduction in Sox4 and Fgfr1 at the protein but not mRNA levels (Figure 4D and Supplemental Figure 3, C and D). A time-course analysis showed that the reduction in both Sox4 and Fgfr1 protein expression was a rapid event that occurred even after 1 hour of exposure to zotatifin (Figure 4E), consistent with the short half-life of Sox4 and Fgfr1 proteins (Supplemental Figure 3E). The human TNBC cell line BT549 also showed a similar pattern of inhibition of SOX4 and FGFR1 at the protein but not RNA levels upon zotatifin treatment (Figure 4F and Supplemental Figure 3, F and G). This effect is not due to a nonspecific stress response, as zotatifin did not increase the phosphorylation of eIF2α, an integrated stress response marker, nor did stress-causing chemotherapeutic drugs result in reduction of Sox4 or Fgfr1 (Supplemental Figure 3H). To further explore whether the observed effects of zotatifin are through targeting eIF4A1, we employed HAP1 cells that express a mutant eIF4A1 (F163L) generated through CRISPR/Cas9-mediated gene editing (23). This mutation can abolish the binding of zotatifin to eIF4A1 but does not affect the function of eIF4A1 (14). Whereas SOX4 and FGFR1 protein but not RNA expression was downregulated by zotatifin in wild-type and Cas9 control (F163F) HAP1 cells, these effects were abrogated in eIF4A1-F163L mutant cells (Figure 4G and Supplemental Figure 3I), demonstrating the specificity of eIF4A1 targeting by zotatifin. Collectively, these data suggest that the reduction in Sox4 and Fgfr1 protein abundance can serve as robust pharmacodynamic biomarkers for zotatifin drug activity.

To further study how zotatifin regulates Sox4 and Fgfr1 protein expression, we performed polysome profiling of 2153L cells treated with either vehicle or a low concentration of zotatifin (40 nM) for 2 hours in vitro (Figure 4H). This low dose and short time period should mitigate any off-target or secondary effects of drug treatment. We found that zotatifin treatment dramatically increased the abundance of 80S monosomes, but only had a modest effect on the abundance of polysomes (Figure 4I), suggesting that zotatifin blocked the translation of a subset of mRNA transcripts. Subsequent qPCR analysis showed that zotatifin did not affect the translation efficiency of housekeeping genes *Actb* and *Gapdh* (Supplemental Figure 3J); in contrast, it reduced the translation efficiency for both *Sox4* and *Fgfr1* transcripts (Figure 4J). Consistently, cycloheximide (CHX) abolished the zotatifin-induced reduction in Sox4 and Fgfr1 proteins (Supplemental Figure 3K), demonstrating that zotatifin inhibits the expression of *Sox4* and *Fgfr1* at the translational level.

### Zotatifin elicits an IFN response through inhibition of Sox4 translation.

**Figure 5:**
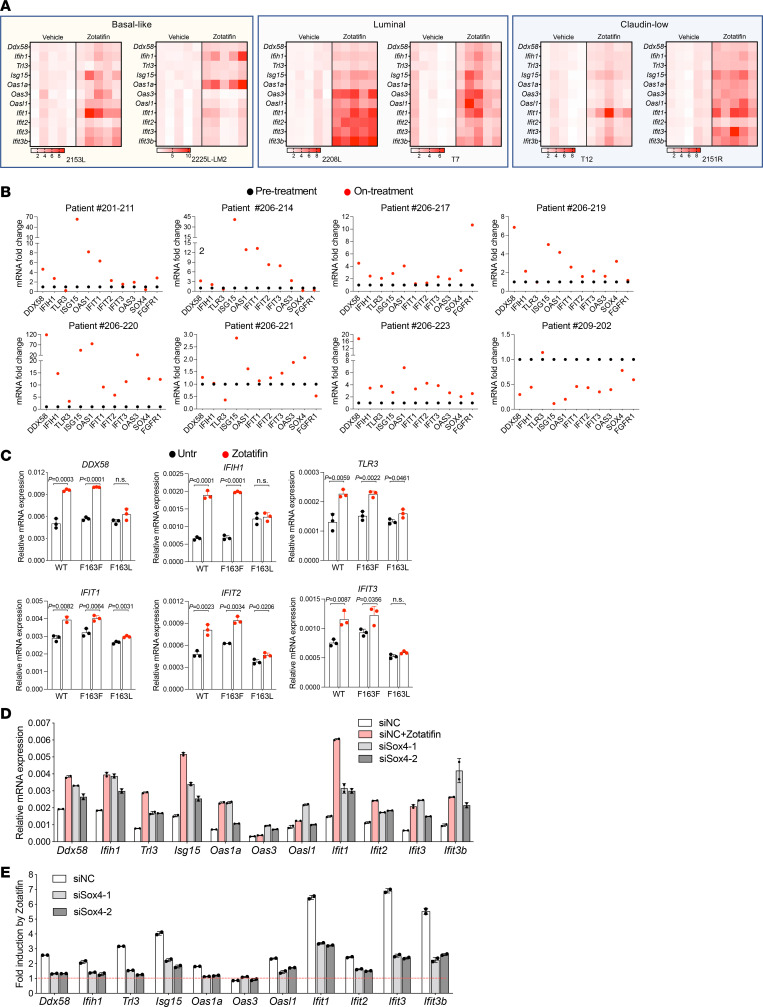
Inhibition of Sox4 translation contributes to zotatifin-induced IFN response. (**A**) qPCR analysis of tumors that were treated with vehicle or zotatifin in vivo. The mean mRNA levels of the vehicle groups were set as 1 and fold changes were calculated for each gene. *n =* 5 biological replicates per group. (**B**) qPCR analysis of 8 paired biopsies from pretreatment (black) and on-zotatifin-treatment (red) ER^+^ breast cancer patients. The mRNA levels of pretreatment samples were set as 1 and fold changes were calculated for each paired sample. (**C**) qPCR analysis of HAP1 cells that were treated with 40 nM zotatifin for 6 hours. Data are representative of 2 independent experiments and are presented as mean ± SD of technical triplicates. (**D**) qPCR analysis of 2153L cells that were transfected with negative control siRNA with or without zotatifin treatment, or *Sox4* siRNAs without zotatifin treatment for 48 hours. (**E**) qPCR analysis of zotatifin-induced gene fold changes in 2153L cells that were transfected with negative control siRNA or *Sox4* siRNAs in the presence of vehicle or zotatifin. In **D** and **E**, representative data from 3 biological replicates are shown, and data are presented as mean ± SD of technical duplicates.

The observation of increased expression of proteins involved in IFN-α and IFN-γ responses upon zotatifin treatment was initially counterintuitive because targeting eIF4A by zotatifin primarily suppresses mRNA translation. Therefore, we hypothesized that the induction of IFN response–related proteins might be a secondary event of eIF4A targeting. This hypothesis was supported by the observation that not only the protein but also the RNA expression of genes such as *Ddx58*, *Ifih1*, and *Tlr3*, which are intracellular pattern recognition receptors (PRRs), and several IFN-stimulated genes (ISGs) were markedly increased upon zotatifin treatment in 2153L tumors (Figure 3B and Figure 5A). This induction was observed in all 6 GEM models in vivo (Figure 5A) and in a dose- and time-dependent manner in vitro (Supplemental Figure 4, A and B). In contrast, several commonly used chemotherapeutic drugs failed to induce ISG mRNAs (Supplemental Figure 4C). The effect of zotatifin is specific to targeting eIF4A1 because the induction of IFN response genes was abrogated in eIF4A1-F163L mutant HAP1 cells (Figure 5C). Furthermore, a subset of biopsy samples from heavily pretreated ER^+^ breast cancer patients treated with zotatifin in ongoing clinical trials (ClinicalTrials.gov NCT04092673) showed induction of many of the ISGs compared with pretreatment (Figure 5B and Supplemental Figure 4D). Given the heterogeneous nature of tumor tissue, these gene expression signals may come from both tumor cells and tumor-infiltrating immune cells. While eIF4A1 is the major zotatifin target (Supplemental Figure 5, A and B), and compared with non-TNBC, TNBCs express higher levels of eIF4A1 at both the RNA and protein levels (Supplemental Figure 5C), unfortunately, no TNBC patients have been included in these trials to date, so a direct comparison with the preclinical data is not possible. Collectively these data indicate that the induction of IFN-response RNAs serves as a robust pharmacodynamic biomarker for zotatifin activity in addition to the reduction in Sox4 and Fgfr1 protein.

Sox4 has been reported to directly suppress the transcription of multiple genes involved in the IFN response (40). Accordingly, inhibiting *Sox4* expression using siRNAs (Supplemental Figure 5, D and E) increased the levels of PRR and ISG mRNAs in 2153L (Figure 5D) and as a positive control BT549 cells (Supplemental Figure 5F), in which ISGs were previously shown to be regulated by Sox4 (40). Importantly, when *Sox4* was inhibited, the ability of zotatifin to induce PRR and ISG expression was partially impaired (Figure 5E and Supplemental Figure 5G). Consistently, zotatifin failed to induce PRR and ISG mRNAs in the Sox4-lacking E0771 cells (Supplemental Figure 5H). These data suggest that zotatifin induces an IFN response at least in part by inhibiting the translation of *Sox4*.

### Zotatifin synergizes with carboplatin in suppressing tumor growth.

**Figure 6:**
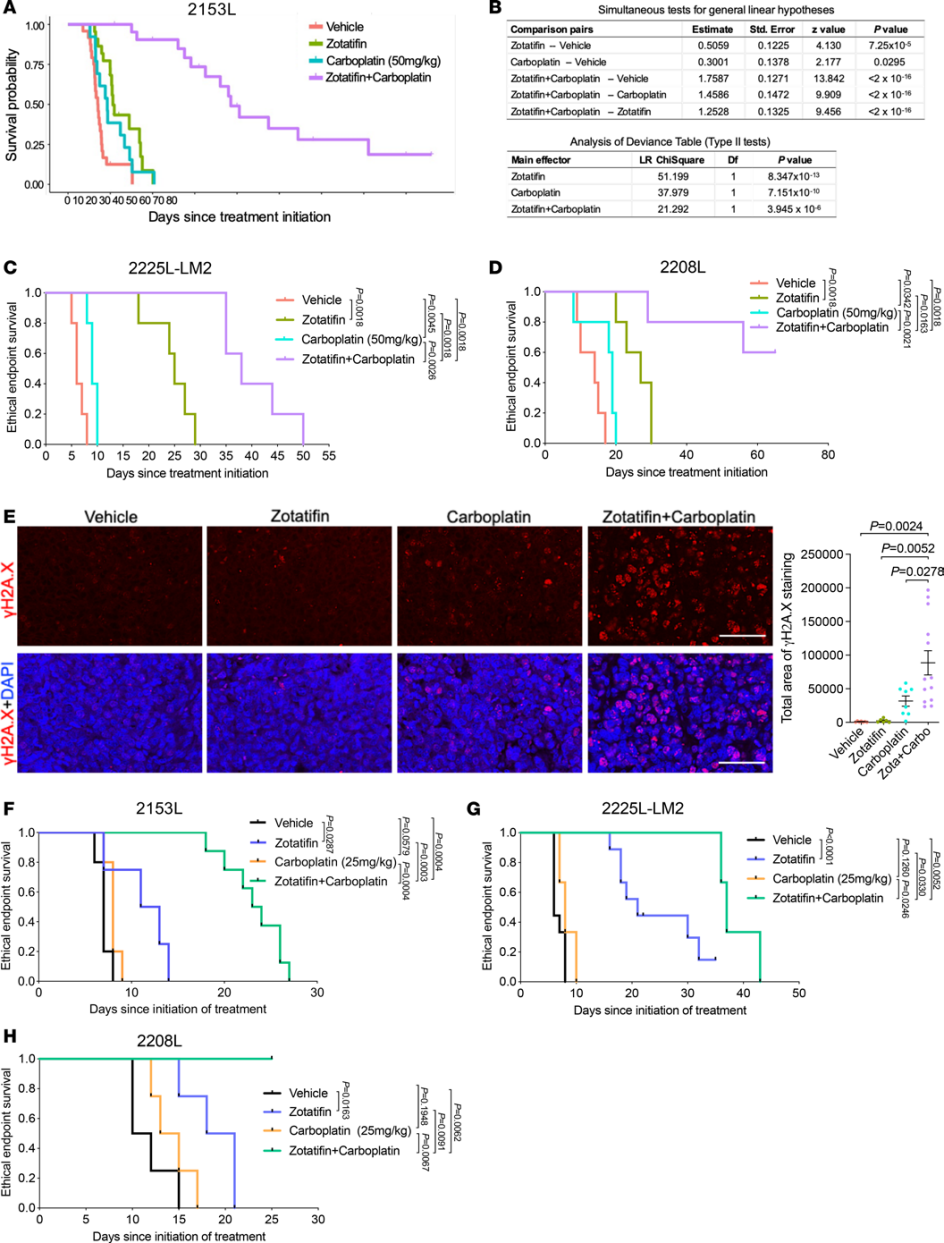
Zotatifin synergizes with carboplatin to suppress tumor progression. (**A**) Kaplan-Meier survival curves of 2153L tumor–bearing mice from treatment start time. (**B**) Survival regression analysis of 2153L tumor–bearing mice. Survival data were fitted using a parametric survival regression model with a log-normal distribution. The top table reports all possible pairwise comparisons using linear contrasts that are adjusted for multiple comparisons using Holm’s method. The bottom table tests for overall main effects and interactions. In **A** and **B**, data from 3 to 5 independent experimental batches are integrated. *n =* 24 for vehicle, *n =* 22 for zotatifin, *n =* 13 for carboplatin, and *n =* 24 for zotatifin + carboplatin. (**C** and **D**) Kaplan-Meier survival curves of 2225L-LM2 (**C**) or 2208L (**D**) tumor–bearing mice from treatment start time. Mice were randomized and treatment was initiated when 2225L-LM2 tumors reached approximately 130 mm^3^ volume or 2208L tumors reached approximately 210 mm^3^ volume. *n =* 5 biological replicates per group. (**E**) Left: Representative images of IF staining of γH2A.X (in red) in 2153L tumors that were treated with indicated drugs for 3 days. Scale bars: 50 μm. Right, quantification of IF staining. At least 2 representative views were analyzed for each tumor and at least 3 tumors for each treatment group were analyzed. Data are presented as mean ± SEM and were analyzed using 2-tailed, unpaired Student’s *t* test. (**F**) Kaplan-Meier survival curves of 2153L tumor–bearing mice treated with indicated drugs. *n* ≥ 4 biological replicates per group. (**G**) Kaplan-Meier survival curves of 2225L-LM2 tumor–bearing mice treated with indicated drugs. *n* ≥ 3 biological replicates per group. (**H**) Kaplan-Meier survival curves of 2208L tumor–bearing mice treated with indicated drugs. *n =* 4 biological replicates per group. In **C**, **D**, and **F**–**H**, the log-rank test (2-tailed) was used to test for the significant differences of curves between groups.

Although zotatifin as a monotherapy was effective in suppressing tumor growth, it did not lead to a durable response. In the clinic, novel targeted therapies will first be tested in combination with standard-of-care therapies. In light of this, we examined whether combining zotatifin with carboplatin, a routinely used chemotherapy for TNBC (41), would be a more effective treatment. For this purpose, we orthotopically transplanted 2153L tumors into the mammary fat pad of BALB/c mice and initiated either monotherapy or combination treatment when tumors reached 120 mm^3^ (Supplemental Figure 6A). The 2153L tumors were minimally sensitive to carboplatin even at the clinically relevant dose (50 mg/kg); however, the addition of zotatifin with carboplatin dramatically inhibited tumor growth in 4 independent experiments (Supplemental Figure 6A) and substantially prolonged survival (Figure 6A). Remarkably, 3 mice exhibited complete tumor regression following combination therapy and remained tumor free for months after the treatment stopped.

To assess whether there was a statistical interaction between these 2 drugs rather than an additive effect of monotherapies, we performed parametric survival regression analysis using the accelerated failure time model (42). This analysis demonstrated that both zotatifin and carboplatin had a positive effect on mouse survival. However, the combination therapy group had much longer survival than expected based on the additive effect of monotherapies (Figure 6B), indicating a strong interaction between zotatifin and carboplatin. It is worth noting that the combination therapy was well tolerated and did not cause body weight loss (Supplemental Figure 6B). The combination therapy produced a much greater survival benefit than monotherapy in the 2225L-LM2 and 2208L tumor models as well (Figure 6, C and D). To investigate the mechanisms responsible for this striking effect, we analyzed 2153L tumors 3 days after drug treatment. At this early treatment stage, zotatifin monotherapy had a minimal effect on cell proliferation and apoptosis, while carboplatin monotherapy minimally inhibited cell proliferation but promoted cell apoptosis (Supplemental Figure 6, C and D). However, the combination therapy not only notably inhibited cell proliferation but also dramatically induced DNA damage, as indicated by the formation of γH2A.X foci (Figure 6E) and enhanced apoptosis (Supplemental Figure 6, C and D). These data suggest a strong synergy between zotatifin and carboplatin.

An important clinical issue is the toxicity of chemotherapies such as neutropenia. If tumors can be sensitized to lower doses of chemotherapy, side effects are likely to be greatly reduced. Therefore, we tested whether zotatifin could confer a therapeutic benefit to carboplatin at half of the clinically relevant dose (25 mg/kg) in 3 GEM models. In 2153L, zotatifin in combination with half-dose carboplatin markedly suppressed tumor growth and prolonged survival compared with monotherapies (Figure 6F and Supplemental Figure 6E). In 2225L-LM2 and 2208L models, the half-dose of carboplatin had a minimal effect on survival, whereas zotatifin monotherapy effectively prolonged survival and strikingly in combination with carboplatin led to an overall improved survival benefit (Figure 6, G and H). We also tested docetaxel, another routinely used chemotherapy for treatment of TNBC, at half the clinically relevant dose (10 mg/kg) in 2153L. While 2153L was completely resistant to low-dose docetaxel, the combination with zotatifin significantly inhibited tumor growth and prolonged mouse survival (Supplemental Figure 6, F and G). These data suggest that zotatifin may effectively sensitize insensitive tumors to low-dose chemotherapies.

### Zotatifin synergizes with carboplatin to induce a heightened IFN response and T cell–dependent durable tumor suppression.

**Figure 7:**
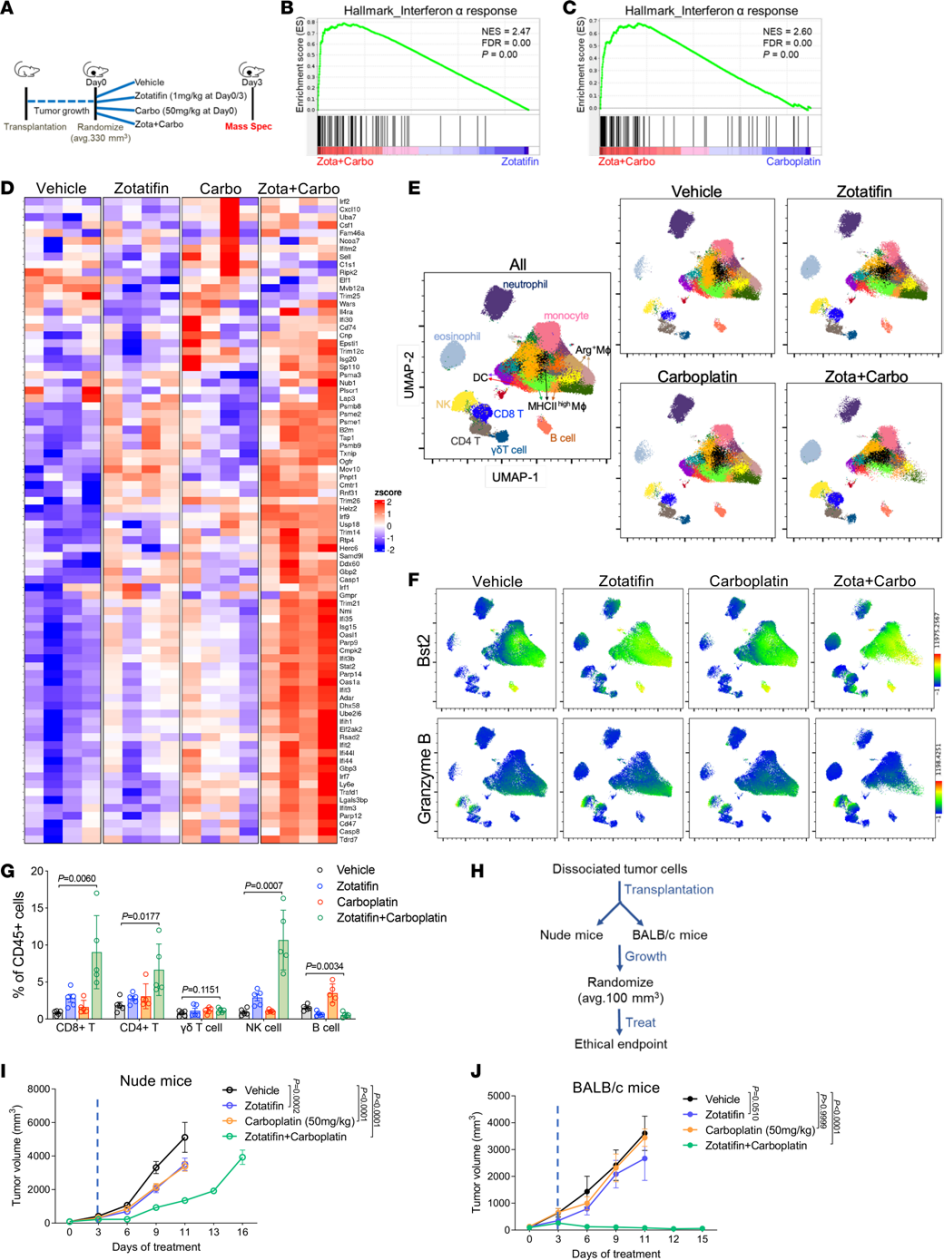
Zotatifin synergizes with carboplatin to induce an IFN response and promote T cell–dependent tumor inhibition. (**A**) Scheme of sample collection strategy for mass spectrometry. Tumor-bearing mice were randomized and treated with indicated therapies for 3 days. Tumor tissues were collected 3 hours after the second injection of zotatifin. *n* = 4 per group. (**B** and **C**) GSEA enrichment plot for Hallmark IFN-α response signature that is upregulated in combination therapy treated tumors compared with zotatifin monotherapy (**B**) or carboplatin monotherapy (**C**). (**D**) Heatmap for Hallmark IFN-α response signature proteins from tumor tissues treated with indicated therapies. (**E**–**G**) Mass cytometry analysis of tumor-infiltrating immune cells of 2153L tumors that were treated with indicated therapies for 7 days. (**E**) The uniform manifold approximation and projection (UMAP) plot overlaid with color-coded clusters. Data from 5 biological replicates of each group were concatenated before UMAP and FlowSOM clustering analysis. Equal numbers of events are shown for each group and major cell types are marked. (**F**) UMAP plot overlaid with the expression of Bst2 or granzyme B. (**G**) Quantification of major lymphoid populations from 2153L tumors in mass cytometry analysis. *n =* 5 biological replicates per group. (**H**) Outline of treatment design. Freshly dissociated 2153L tumor cells were transplanted into nude mice or BALB/c mice in parallel. Treatment was initiated when tumors reached 100 mm^3^. (**I**) Growth curves of 2153L tumors in nude mice treated with indicated drugs. *n =* 5 biological replicates per group. (**J**) Growth curves of 2153L tumors in BALB/c mice treated with indicated drugs. *n =* 3 biological replicates for monotherapy groups and *n =* 10 biological replicates for the combination treatment group. In **I** and **J**, data are presented as mean ± SEM and were analyzed using 2-way ANOVA with Bonferroni’s multiple-comparison test.

To investigate the mechanisms underlying the synergistic effect of zotatifin and carboplatin, we conducted proteomic analysis of 2153L tumors treated with either monotherapy or combination therapy for 3 days in vivo (Figure 7A). Interestingly, although zotatifin alone induced a robust IFN response (Figure 3, C and E), the combination with carboplatin further markedly increased the IFN response as compared with either monotherapy, as revealed in GSEA (Figure 7, B and C, and Supplemental Tables 4 and 5). Combination therapy increased both the number and the level of expression of induced IFN pathway proteins (Figure 7D). Interestingly, this synergy was not observed in vitro, as combination treatment did not lead to a greater induction of IFN response genes compared with zotatifin alone (Supplemental Figure 7A), suggesting that the tumor immune microenvironment contributed to the response. Therefore, we performed mass cytometry of dissociated 2153L tumors and included in the panel Bst2, an IFN-stimulated transmembrane protein (43). We observed increased Bst2 expression in almost all the major immune cell populations upon zotatifin monotherapy and importantly, to a greater degree in combination-therapy tumors (Figure 7, E and F, and Supplemental Figure 7B). These data suggest that zotatifin and combination therapy not only induced an IFN response in tumor cells but also in tumor-infiltrating immune cells.

**Figure 8:**
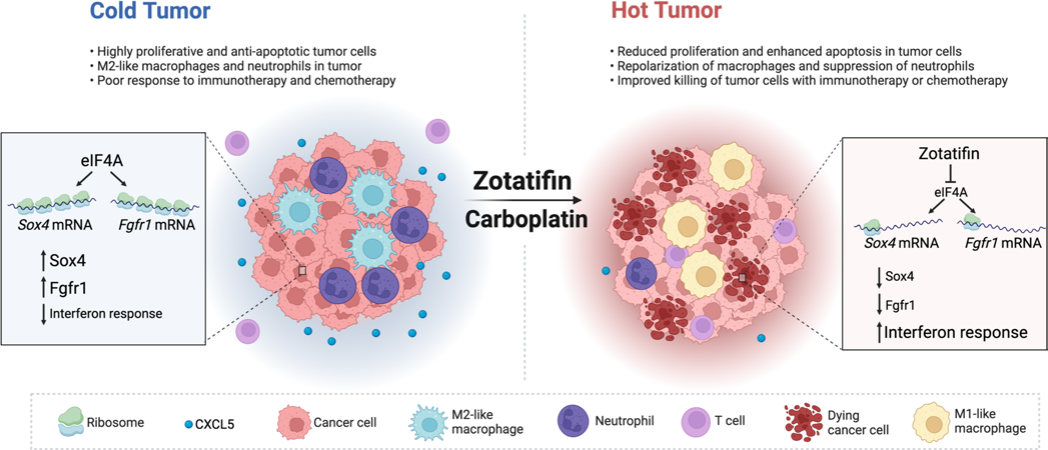
Working model of zotatifin. Inhibition of eIF4A by zotatifin suppresses the translation of *Sox4* and *Fgfr1*, induces an IFN response, shifts the tumor immune landscape, and ultimately enhances the response to immune checkpoint blockade or chemotherapy.

Combination therapy elicited dramatic changes in both the myeloid and lymphoid compartments of the tumor microenvironment, including decreased infiltration of neutrophils and Arg1^+^ macrophages and increased infiltration of eosinophils, NK cells, CD8^+^ T cells, and CD4^+^ T cells (Figure 7, E and G, and Supplemental Figure 7B). In addition, many of the NK cells and CD8^+^ T cells exhibited granzyme B expression (Figure 7F). The increased infiltration of CD4^+^ and CD8^+^ T cells was also confirmed with immunostaining (Supplemental Figure 8, A and B). To investigate whether T cell immunity played a role in the sustained tumor inhibition by combination therapy, the same number of freshly dissociated 2153L tumor cells were transplanted in parallel into immunocompetent BALB/c mice and T cell–deficient athymic nude mice. Treatment was initiated when tumors reached 100 mm^3^ (Figure 7H). In nude mice, zotatifin and carboplatin monotherapies slowed tumor growth, and combination therapy initially decreased tumor growth for 6 days but was unable to prevent tumor growth beyond this point, and tumors reached the ethical endpoint by day 16 (Figure 7I). In contrast, although monotherapies showed only mild effects in BALB/c mice, the combination therapy elicited marked tumor regression after day 3, which continued at least until day 15 (Figure 7J). These data suggest that while T cells may not affect the efficacy of monotherapy, they contribute to the durable response elicited by combination therapy. Taken together, these findings suggest that inhibition of eIF4A by zotatifin reprograms the translatome, shifts the tumor immune landscape, and ultimately enhances the response to ICB or chemotherapy (Figure 8).

## Discussion

The RNA helicase eIF4A is a key node where oncogenic signaling pathways converge to impact cancer progression. The present studies demonstrate that targeting eIF4A by zotatifin can robustly inhibit the translation of *Sox4* and *Fgfr1* in TNBC GEM models, resulting in not only the inhibition of cell proliferation, but also the induction of IFN-related pathways and remodeling of the tumor immune microenvironment. The synergism with carboplatin is exciting, as chemotherapies that encompass taxanes, anthracyclines, and platinums remain the standard of care for patients with TNBC. Importantly, the inhibition of *SOX4* and *FGFR1* translation by zotatifin was observed in both mouse tumors and human breast cancer cell lines, indicating the conserved regulation of these genes by eIF4A. The upregulation of a number of ISGs in on-treatment breast cancer patient biopsy samples further suggests that these findings may be translatable from mouse preclinical models to patients.

Mammals possess 2 highly homologous eIF4A paralogs, eIF4A1 and eIF4A2, that share 90% identity of amino acid sequence and both are targets of rocaglates. Although functionally similar, they are not redundant. In general, eIF4A1 is much more abundant than eIF4A2 (8), which was also observed in the GEM models used in this study and in human breast cancers from the Clinical Proteomic Tumor Analysis Consortium (CPTAC) (Supplemental Figure 5, A and C) (44). Interestingly, eIF4A1, but not eIF4A2, is a common essential gene in most cancer cell lines in CRISPR knockout screens (Supplemental Figure 5B) (45). Besides eIF4A1 and eIF4A2, a recent study identified DDX3X as another target of RocA (46). Similar to eIF4A1/2, DDX3X is a DEAD-box RNA helicase that has been shown to assist translation initiation in yeast, although its function in translation initiation in mammals is more elusive (47). It is important to note that rocaglate treatment may not phenocopy the loss of eIF4A activity (13, 48), probably because of its unique mechanism of action. In the case of all the 3 targets, RocA can increase the RNA affinity of target proteins and clamp the targets onto polypurine RNA in an ATP-independent manner, which inhibits protein synthesis from polypurine-driven reporter mRNAs (46). Importantly, supplementation of recombinant target proteins into an in vitro translation reaction further enhanced RocA-mediated translational repression of polypurine reporter mRNA, indicating a dominant-negative mechanism of action of RocA (15, 46). In contrast, translation inhibition by hippuristanol, which decreases the affinity between eIF4A and RNA and thus mimics the loss of eIF4A function, was relieved by supplementation with recombinant eIF4A (18, 19). Because RocA converts its targets into dominant-negative repressors, the abundance of its target proteins should predict RocA sensitivity, which has been shown in a few cancer cell lines (46), although this remains to be validated using a larger sample size. Furthermore, it is likely that other genetic and epigenetic differences will affect the treatment response. Clinically, breast cancer is divided into different subtypes based on the expression of different steroid receptors and HER2 as well as the PAM50 gene signature (1). Upon interrogating zotatifin target abundance in different breast cancer subtypes, we found that the protein level of EIF4A1 is higher in TNBC as compared with non-TNBC (Supplemental Figure 5C). This suggests that TNBCs may be more sensitive to zotatifin than receptor-positive breast cancers. The current ongoing clinical trials for zotatifin in breast cancer (ClinicalTrials.gov NCT04092673) have been primarily in the context of endocrine resistance in heavily pretreated ER^+^ patients. These results provide the rationale for testing zotatifin especially in combination with chemotherapy or immunotherapy in future clinical trials in patients with TNBC.

Structural analysis revealed that rocaglates target a bimolecular cavity formed by eIF4A and polypurine RNA and Phe163 (F163) in eIF4A1 is crucial for this interaction (14). The specificity of rocaglates for eIF4A has been extensively addressed by multiple studies. A rocaglate-resistant eIF4A1 mutant (F163L) has been characterized, and introduction of this allele into cells using CRISPR/Cas9-mediated gene editing confers resistance to rocaglate cytotoxicity (14, 23, 49, 50). In this study, the nearly complete abrogation of the effects of zotatifin on SOX4/FGFR1/IFN response genes in eIF4A1-F163L HAP1 cells demonstrated the specificity of zotatifin for eIF4A1 (Figure 4G and Figure 5C).

The mechanism of action of rocaglates imposes sequence selectivity onto the general translation factor eIF4A and mRNAs that contain polypurine stretches are more prone to rocaglate-induced repression of mRNA translation. This selectivity is important because it can potentially lead to lower toxicity and better tolerance in animals and humans. Our observation that only a subset of proteins showed altered translation efficiency in response to zotatifin in polysome profiling (Figure 4, I and J, and Supplemental Figure 3J) further attests to the drug selectivity. Moreover, as normal mouse mammary glands express much lower levels of eIF4A1 than GEM models (Supplemental Figure 5A), this further provided a window of selectivity that contributed to the tolerability of zotatifin in mice. In the ongoing clinical trial in ER^+^ breast cancer patients, zotatifin in combination with either fuvelstrant or fulvestrant and abemaciclib were well tolerated, with most adverse effects being grade 1/2 (51). This favorable safety profile is important for its continuous clinical development in potentially other types of cancer. The effect of rocaglates on tumor cells has been characterized in several studies (10, 12, 13, 20, 21), but their effects on the tumor immune microenvironment remain largely unexplored. An important outcome of our study is the observation of the pleiotropic effects of zotatifin, impacting both tumor cells and tumor-infiltrating immune cells. This illustrates the importance of drug testing in preclinical immunocompetent hosts. Our syngeneic GEM models provide a resource of immunocompetent animals for these types of therapeutic studies. Comparative oncogenomics and gene profiling have “credentialed” these models and demonstrated that the resulting mammary tumors are representative of the corresponding human breast cancer subtypes (25–27). In addition, we have demonstrated the utility of these preclinical models not only to study single targeted agents, but also to investigate combination treatments with chemotherapy and immunotherapy. A recent study also examined the direct effects of a few rocaglates, including zotatifin, on primary human immune cells and observed various effects (52). Interestingly, rocaglates have been shown to increase macrophage resistance to *Mycobacterium tuberculosis* infection by directing macrophage polarization toward the M1-like phenotype (53). This is consistent with the results demonstrating the effects of zotatifin on macrophages both in vitro (Figure 2, G–I) and in vivo (Figure 2F). Regarding the mechanism of macrophage polarization, we observed increased Bst2 expression in TAMs upon zotatifin treatment by mass cytometry (Figure 7, E and F). As Bst2 is a type I IFN biomarker (43), we speculated that an induced type I IFN response promoted the polarization of macrophages toward an M1-like phenotype, as reported in multiple studies (54–57). However, this hypothesis awaits further functional studies using *Ifnar1*/*2*–knockout macrophages. Due to their immunosuppressive effects, tumor-infiltrating macrophages have been a target for the development of many experimental drugs. Dual targeting of both tumor cells and macrophages is an important property of zotatifin. Recently, SOX4 was reported to be a critical transcription factor driving the dysfunction of CAR T cells and disruption of SOX4 improved CAR T effector function (58). Given the uniform and potent downregulation of SOX4 by zotatifin treatment in multiple independent tumor models and cell lines, it will be interesting to explore the possibility that zotatifin might potentiate CAR T therapy.

The dramatic synergism between zotatifin and carboplatin is exciting and intriguing. Silvestrol has been shown to enhance sensitivity to doxorubicin by inducing apoptosis in mouse lymphoma models driven by PTEN inactivation or eIF4E overexpression (13). Silvestrol derivatives have also been shown to suppress the expression of CDC6 and induce DNA damage and cell death in a fibrotic pancreatic tumor xenograft model (21). Our study demonstrated that zotatifin synergizes with carboplatin in mounting a heightened type I IFN response. The type I IFN response can drive growth arrest and apoptosis (59). Indeed, increased DNA damage and cell death was observed after 3 days of treatment with zotatifin and carboplatin combination therapy (Figure 6E and Supplemental Figure 6D). It is likely that this effect is tumor cell autonomous, as T cell immunity might not have occurred at this early stage. The similar tumor growth kinetics observed in nude and BALB/c mice before day 6 support this hypothesis (Figure 7, I and J). In contrast to the early response, durable tumor suppression by combination therapy was observed only in BALB/c mice, indicating that T cells contribute to the long-term therapeutic effect. Type I IFN signaling bridges the innate and adaptive immune responses and contributes to therapeutic efficacy (60, 61). Thus, it is possible that the robust IFN response induced by combination therapy in these studies promoted T cell immunity. Besides carboplatin, zotatifin also synergized with docetaxel (Supplemental Figure 6, F and G), a non–DNA-damaging chemotherapy routinely used in combination with carboplatin for TNBC treatment (41). It remains to be determined whether zotatifin in combination with docetaxel will trigger disparate immune responses and whether zotatifin in combination with both carboplatin and docetaxel will provide greater therapeutic benefit.

Our study has some limitations. First, we recognize that the transplanted syngeneic TNBC models used in the study may not fully capture the complex nature of tumorigenesis over time and its influence on the immune system. Currently it is not feasible to obtain sufficient numbers of mice with size-matched tumor cohorts using autochthonous spontaneous tumor model to study the effects of different therapies, both singly and in combination. However, we designed our preclinical studies to include models representing the different molecular subtypes of TNBC and started treatment when tumors were approximately 100 mm^3^ in size. Second, we recognize that knockdown of *Sox4* does not fully phenocopy the effect of zotatifin in inducing ISG expression. Considering the complexity of zotatifin action, other mechanisms in addition to *Sox4* repression may control ISG abundance. Thirdly, although we showed that both tumor cells and immune cells exhibited an induced IFN response upon zotatifin treatment, we recognize that the source of ISGs in heterogeneous tumor tissues can only be fully deconvoluted with single-cell RNA sequencing and spatial transcriptomics. Finally, although we identified a few pharmacodynamic biomarkers for zotatifin, the predictive biomarkers for zotatifin sensitivity remain to be identified.

In summary, strategies to augment ICB and chemotherapy are needed for TNBC treatment. The present studies have identified pharmacodynamic biomarkers for zotatifin and provided additional preclinical insight into the mechanisms of zotatifin action both as a single agent and in combination therapies. These studies have provided an important foundation for submission of an investigational new drug application to the FDA to facilitate the next stage of clinical trials for TNBC and potentially other cancer types that also require chemotherapy. Hopefully, these results will help inform future clinical trials and positively impact the treatment of cancer patients.

## Methods

### Tumor transplantation and treatment.

Mice were housed in a room with a 14-hour light/10-hour dark cycle at a temperature of 68°F to 72°F and a humidity of 30% to 70%. The *Trp53*-null GEM TNBC models were previously generated by transplantation of *Trp53*-deleted donor mammary epithelium into the cleared mammary fat pad of syngeneic BALB/c hosts, which gave rise to a cohort of heterogeneous and retransplantable mammary tumors (24). For tumor transplantation, GEM tumors were first freshly dissociated into single cells as previously reported (62). Briefly, tumor tissues were digested in 1 mg/mL collagenase A (Sigma-Aldrich, 11088793001) for 2 hours at 37°C with 125 rpm rotation followed by a brief centrifugation to enrich tumor organoids. Tumor organoids were subsequently trypsinized and filtered into single cells. Then, 25,000 tumor cells were transplanted into the fourth mammary fat pad of 6- to 8-week-old female BALB/c mice (Envigo, 047) or athymic nude mice (Hsd:Athymic Nude-*Foxn1^nu^*; Envigo, 069). For cell line models 4T1 and E0771, 200,000 tumor cells were transplanted into the fourth mammary fat pad of 6- to 8-week-old female BALB/c mice or C57BL/6 mice (Jackson Laboratory, 000664), respectively. When tumors reached an average size of 70 to 150 mm^3^, mice were randomized followed by drug treatment. Tumor volume and body weight were measured every 2 to 3 days. Investigators were not blinded to the group assignment. Tumor volume was calculated as (length × width^2^)/2. The ethical endpoint was met when the tumor reached a volume of 1500 mm^3^. For BrdU labeling of proliferative cells, mice were injected with 60 mg/kg BrdU (Sigma-Aldrich, B-5002) 2 hours before sacrifice.

The following drugs and dosages were used in this study: Zotatifin (eFT226, eFFECTOR Therapeutics) was dissolved in 5% dextrose (Sigma-Aldrich, G7528) and dosed at 1 mg/kg every 3 days. Carboplatin (Sigma-Aldrich, C2538) was dissolved in PBS and administered at 25 or 50 mg/kg weekly. Docetaxel (LC Laboratories, D-1000) was first dissolved in Tween 80 and then diluted 1:4 with 16.25% ethanol and administered at 10 mg/kg weekly. IgG (BioXCell, BE0089 and BE0086), anti–PD-1 (BioXCell, BE0146), and anti–CTLA-4 (BioXCell, BE0164) were diluted in PBS and dosed at 10 mg/kg every 3 days. Anti-Csf1r (Syndax Pharmaceuticals, SNDX-ms6352) was dosed at 40 mg/kg for the initial dose followed by 20 mg/kg for the remaining dose every week. All drugs and vehicles were administered via intraperitoneal injections.

### Primary cell line generation and cell culture.

To establish the primary 2153L cell line, single cells isolated from 2153L tumor tissues were cultured in medium containing 50 μg/mL G418 (Thermo Fisher Scientific, 10131035) for 2 weeks to deplete stromal cells, because the genetic engineering event that led to the deletion of *Trp53* alleles in *Trp53*-null GEM models involved a neomycin resistance gene. Established 2153L cells were cultured in DMEM/F-12 medium (Thermo Fisher Scientific, 11330032) supplemented with 10% fetal bovine serum (FBS) (GenDEPOT, F0900-050), 5 μg/mL insulin (Sigma-Aldrich, I-5500), 1 μg/mL hydrocortisone (Sigma-Aldrich, H0888), 10 ng/mL epidermal growth factor (Sigma-Aldrich, SRP3196), and 1× Antibiotic-Antimycotic (Thermo Fisher Scientific, 15240062). 2153L cells were tested to be free of mycoplasma contaminants using the Universal Mycoplasma Detection Kit (ATCC, 30-1012K). BT549 cells were acquired from ATCC and cultured in RPMI-1640 (GenDEPOT, CM058-050) supplemented with 10% FBS and 1× Antibiotic-Antimycotic. 4T1 and E0771 cells were acquired from Xiang Zhang’s laboratory at the Baylor College of Medicine (28) and cultured in DMEM (GenDEPOT, CM002-050) supplemented with 10% FBS and 1× Antibiotic-Antimycotic. HAP1 cells were acquired from eFFECTOR therapeutics (23) and cultured in IMDM (Thermo Fisher Scientific, 12440046) supplemented with 10% FBS and 1× Antibiotic-Antimycotic. The following drugs were used for cell culture treatment: CHX (Sigma-Aldrich, C4859) at 100 μg/mL, MG132 (MedChemExpress, HY-13259) at 50 μM, docetaxel (LC Laboratories, D-1000), camptothecin (MedChemExpress, HY-16560), and doxorubicin (LC Laboratories, D-4000).

### Patient biopsy samples.

Deidentified frozen core biopsy samples were obtained from 8 ER^+^ breast cancer patients who were heavily pretreated and then enrolled in the zotatifin phase I/II clinical trial (ClinicalTrials.gov NCT04092673). Tumor tissues came from either primary (for patient 201-211) or metastatic (for the additional 7 patients) sites. All the patients received 0.07 mg/kg zotatifin on a 2-week on/1-week off schedule. Patient 206-214 received zotatifin monotherapy and the additional 7 patients received zotatifin in combination with fulvestrant. Paired pretreatment and on-zotatifin treatment biopsies were acquired from each patient, and on-treatment specimens were collected at C1D9 (approximately 24 hours after the second dose of zotatifin).

### Immunohistochemistry and immunofluorescence.

Tumor tissue specimens were fixed in 4% paraformaldehyde overnight and stored in 70% ethanol until paraffinization and embedding. Tissue sections of 5 μm thickness were deparaffinized, rehydrated, and subjected to antigen retrieval in citrate buffer (pH 6.0, Sigma-Aldrich, C9999) for 20 minutes in a steamer. Slides were incubated with primary antibodies overnight at 4°C and with secondary antibodies for 1 hour at room temperature before ABC-HRP (Vector Laboratories, PK-7100) treatment and DAB (Vector Laboratories, sk-4105) development for immunohistochemistry (IHC) or DAPI staining for immunofluorescence (IF). The antibodies and dilutions used were as follows: anti-F4/80 (1:1000; Cell Signaling Technology [CST], 70076S), anti-S100A8 (1:5000; R&D Systems, MAB3059), anti-BrdU (1:1000; Abcam, ab6326), anti–cleaved caspase 3 (1:1000; CST, 9661), anti–phospho-histone H2A.X (Ser139) (1:400; CST, 9718), anti-CD4 (1:800; Abcam, ab183685), and anti-CD8 (1:800; eBioscience, 14-0808-82).

All IHC slides were scanned with an Aperio ImageScope (Leica Biosystems) and analyzed using Aperio ImageScope software (v12.3.3.5048). At least 3 representative ×20 views were analyzed for each tumor and at least 3 tumors for each treatment group were analyzed. Specifically, the percentage of BrdU^+^, cleaved caspase 3^+^, or CD4^+^ cells was analyzed using the Aperio Nuclear v9 algorithm. The total percentage of positive nuclei was counted (including strongly, moderately, and weakly stained cells) for BrdU^+^ and cleaved caspase 3^+^ cells, and only the strongly stained signals were counted for CD4^+^ cells. To calculate the percentage of S100A8^+^ or CD8^+^ cells, positively stained cells were counted manually to avoid the inclusion of background staining. This number was then divided by the total cell number for each view, which was automatically counted using the Aperio Nuclear v9 algorithm. IF images were taken using a Nikon A1-Rs confocal microscope at ×40 magnification and analyzed using Python v3.8.15 (https://www.python.org/downloads/release/python-3815/). Specifically, the γH2A.X channel mask was obtained by first applying the MaxEntropy thresholding filter followed by applying a binary closure with Gaussian filter (σ = 2). Any object smaller than 9 pixels was removed to obtain the final mask. At least 2 representative views were analyzed for each tumor and at least 3 tumors for each treatment group were analyzed.

### Polysome profiling analysis.

Sucrose gradients (10%–50%) were prepared in polysome buffer (20 mM Tris pH 7.4, 150 mM NaCl, 5 mM MgCl_2_, 1 mM DTT, and 100 μg/mL CHX) supplemented with 20 U/mL SUPERaseIn RNase Inhibitor (Invitrogen, AM2696) and RNase-free sucrose (Sigma-Aldrich, 84097), poured into polypropylene tubes (Beckman Coulter, 331374) the evening before use, and stored at 4°C.

2153L cells were seeded in 15 cm plates overnight and reached 80% confluence on the day of harvest. Cells were treated with DMSO (0.0004%) or 40 nM zotatifin for 2 hours before incubation in 100 μg/mL CHX for 5 minutes. Next, cells were washed with PBS and trypsinized to single cells in the presence of 100 μg/mL CHX, followed by washing in ice-cold PBS with CHX and lysing in 500 μL of ice-cold lysis buffer (polysome buffer supplemented with 1% Triton X-100 and 25 U/mL Turbo DNase I [Invitrogen, AM2238]). Cell lysates were triturated 5 times through an 18-gauge needle and 10 times through a 27-gauge needle, followed by incubation on ice for 10 minutes. Then, lysates were cleared by centrifugation for 10 minutes at 14,000*g* and 4°C, and an equal volume of supernatant was carefully layered on top of the sucrose gradients. The gradients were ultracentrifuged at 35,000 rpm for 2 hours at 4°C using an SW41 Ti rotor (Beckman Coulter). The gradients were then displaced into a UA-6 continuous UV detector (Teledyne ISCO) using a syringe pump (Brandel) containing Fluorinert FC-40 (Sigma-Aldrich, F9755) at a speed of 0.75 mL/min. Absorbance was recorded at an OD of 260 nm using the Logger Lite (v1.9.4) software (Vernier). A total of 24 fractions with 500 μL volume were collected for each sample using the Foxy Jr fractionator.

Two hundred fifty microliters of each fraction was mixed with 500 pg of luciferase RNA spike-in (Promega, L4561) and lysed in 750 μL TRIzol LS (Thermo Fisher Scientific, 10296028). RNA was precipitated in the presence of GlycoBlue (Invitrogen, AM9515) and dissolved in 20 μL RNase-free water. Then, 6 μL of RNA from each fraction was converted to cDNA using the High-Capacity cDNA Reverse Transcription Kit (Thermo Fisher Scientific, 4368814). The cDNA was diluted 10 times before qPCR analysis. The qPCR data were processed as previously reported (63).

### Statistics.

Unpaired, 2-tailed Student’s *t* tests were performed to compare the differences between 2 groups in most studies where indicated. For proteomics data, differential analysis was performed using the moderated *t* test, as implemented in limma (64). Two-way ANOVA with Bonferroni’s or Tukey’s multiple-comparison test was used to analyze tumor volume or body weight over time. The log-rank test was used to test for significant differences in the Kaplan-Meier survival curves between groups for Figure 2K, Figure 6, C, D, and F–H, and Supplemental Figure 6G. Mice whose tumors had never reached the target size were censored at their last time point. Censored events are marked as vertical ticks on the curves. All above analyses were performed using GraphPad Prism 9 software. The survival regression analysis shown in Figure 6, A and B was performed using R software (https://www.r-project.org/). For these data, the time to the endpoint tumor size (1500 mm^3^) for each mouse was computed by linear interpolation using the 2 time points and tumor sizes immediately before and immediately after crossing the boundary for the first time. Survival data were modeled using a parametric survival regression based on a log-normal distribution with main effects for each treatment and their interaction. All possible pairwise comparisons were computed using linear contrasts and adjusted for multiple comparisons using Holm’s method. All *P* values were 2-sided. A *P* value of 0.05 or less was considered significant.

### Study approval.

All mouse studies were conducted in compliance with the NIH *Guide for the Care and Use of Laboratory Animals* (National Academies Press, 2011). All mice were maintained and euthanized according to the guidelines of IACUC at Baylor College of Medicine (protocol AN-504). The deidentified patient biopsy study was reviewed and approved by the IRB at Baylor College of Medicine and was considered as a human material study.

### Data availability.

All data generated in this study are available within the article, its supplemental information, the Supporting Data Values file, and from the corresponding author upon reasonable request. All raw mass spectrometry data were deposited in the publicly available MassIVE/ProteomeXchange under accession number MSV000089580/PXD034250.

## Author contributions

NZ conceived the study, designed and conducted the experiments, analyzed the data, and wrote the manuscript. EBK, XY, LCR, NL, YG, DAP, SJC, AJS, and CH performed experiments. ABS and AM designed the mass spectrometry experiments, analyzed the data, and edited the manuscript. KS analyzed the IF images. SS and BZ analyzed the CPTAC data. JZ and LMS scanned immunostaining slides using the Aperio system. SGH performed biostatistical analyses. CF performed bioinformatics analyses. CMP performed bioinformatics analyses and edited the manuscript. JMR conceived and supervised the study and edited the manuscript.

## Supplementary Material

Supplemental data

Supplemental table 1

Supplemental table 2

Supplemental table 3

Supplemental table 4

Supplemental table 5

Supplemental table 6

Supporting data values

## Version 1 10/24/2023

In-Press Preview

## Version 2 12/15/2023

Electronic publication
