# Supplementary material for: Targeting eIF4A triggers an interferon response to synergize with chemotherapy and suppress triple-negative breast cancer
Source: J Clin Invest. 2023 Dec 15;133(24):e172503. doi: 10.1172/JCI172503 (PMC10721161; doi:10.1172/JCI172503)

Supplementary materials

Title: Targeting EIF4A triggers an interferon response to synergize with chemotherapy and suppress triple-negative breast cancer

Authors: Na Zhao[1], Elena B. Kabotyanski[1], Alexander B. Saltzman[2], Anna Malovannaya[2,3], Xueying Yuan[1], Lucas C. Reineke[4], Nadia Lieu[1], Yang Gao[1], Diego A Pedroza[1], Sebastian J Calderon[1], Alex J Smith[1], Clark Hamor[1], Kazem Safari[5], Sara Savage[6], Bing Zhang[6], Jianling Zhou[7], Luisa M. Solis[7], Susan G. Hilsenbeck[6], Cheng Fan[8], Charles M. Perou[8], Jeffrey M. Rosen[1*]

Affiliations:

1. Department of Molecular and Cellular Biology, Baylor College of Medicine, Houston, Texas, USA.
2. Mass Spectrometry Proteomics Core, Baylor College of Medicine, Houston, Texas, USA.
3. Department of Biochemistry and Molecular Pharmacology, Baylor College of Medicine, Houston, Texas, USA.
4. Department of Neuroscience, Baylor College of Medicine, Houston, Texas, USA.
5. Texas A&M Health Science Center, Houston, Texas, USA.
6. Lester and Sue Smith Breast Center, Baylor College of Medicine, Houston, Texas, USA.
7. Department of Translational Molecular Pathology, The University of Texas MD Anderson Cancer Center, Houston, Texas, USA.
8. Lineberger Comprehensive Cancer Center, University of North Carolina, Chapel Hill, North Carolina, USA.

* Correspondence to Jeffrey Rosen. E-mail: jrosen@bcm.edu

List of Supplementary Materials

Materials and Methods

References

Supplementary Fig.S1 to S8

Supplementary Table S1 to S6

Materials and Methods

Mass cytometry and flow cytometry

Trp53-null tumors treated with vehicle or Zotatifin were dissociated in 1 mg/ml Collagenase A for 2 hrs at 37°C with 125 rpm rotation followed by 3 short centrifugations to enrich for tumor stromal cells from supernatants. Red blood cells were removed using RBC lysis buffer (Biologend #420301). The remaining single cell suspension was analyzed using mass cytometry or flow cytometry.

In mass cytometry, after staining with cisplatin viability dye, cells were surface stained using a cocktail of antibodies conjugated to metal isotopes, fixed, permeabilized using Foxp3 staining buffer set (eBioscience #00552300), and stained with antibodies for intracellular markers. The cells were then stained with Cell-ID Intercalator-Ir (Fluidigm #SKU201192A) overnight at 4°C and analyzed using a Helios CyTOF Mass Cytometer (Fluidigm). The normalized FCS files were first processed using FlowJo to remove beads, dead cells, and doublets. Equal numbers of CD45+ single cells from biological replicates of each treatment group were concatenated and subjected to analysis in Cytobank. Data were dimensionally reduced using viSNE (https://search.r-project.org/CRAN/refmans/CytobankAPI/html/viSNE-class.html) and cell clusters were identified using FlowSOM (https://bioconductor.org/packages/release/bioc/html/FlowSOM.html).

For flow cytometry of tumor-infiltrating immune cells or BMDMs, single cells were stained with LIVE/DEAD Fixable Yellow Dead Cell Stain (Thermo Fisher #L34967), blocked with anti-CD16/32 (Biolegend #101330), and stained with surface and intracellular markers according to manufacturer's instructions of the Foxp3 staining buffer set. The following antibodies were used for flow cytometry: CD45 (Biolegend #103128), CD11b (Biolegend #101227), CD206 (Biolegend #141732), Ly-6C (Biolegend #128017), Ly-6G (Biolegend #127651), F4/80 (Biolegend #123115), iNOS (Miltenyi Biotec #130116421), and Arginase (Invitrogen #12369782).

For cell cycle analysis, 2153L cells cultured in regular medium in vitro were allowed to grow to 30% confluence and treated with 40 nM Zotatifin for 48 hrs before trypsinization and fixation in 4% PFA. Next, the cells were

pelleted, washed with PBS, and resuspended in DAPI staining buffer (Thermo Fisher #R37606) before flow cytometry.

All flow cytometry data were acquired using an Attune NxT Flow Cytometer (Thermo Fisher) and analyzed using FlowJo software (version 10.7.1). The cell cycle distribution was analyzed using the Watson Pragmatic algorithm provided by FlowJo.

Luminex cytokine analysis

2153L tumor tissues from the same batch with TMT MS were pulverized to powder under liquid nitrogen and lysed in MILLIPLEX MAP Lysis buffer (Millipore #43040). Lysates were cleared by centrifugation at 10,000 g three times at 4°C. The protein concentration of the supernatant was measured using a BCA Protein Assay Kit and adjusted to 1 mg/ml using lysis buffer. The abundance of cytokines was determined using a MILLIPLEX Mouse 32-Plex Cytokine Panel 1 kit (Millipore # MCYTMAG-70K-PX32) according to the manufacturer's instructions.

Separation of tumor-associated macrophage (TAM)

Untreated 2153L and 2151R tumors were dissociated in 1 mg/ml Collagenase A for 2 hrs at 37°C and TAMs were separated using EasySep™ Mouse F4/80 Positive Selection Kit (STEMCELL technologies, #100-0659) following manufacture's protocol. TAMs were cultured in BMDM culture medium and treated with either vehicle or 40 nM Zotatifin for 24 hrs before immunoblotting.

Immunoblotting assay

Tumor tissues were snap frozen upon harvest and homogenized in lysis buffer (Tris-HCl pH 6.8, 62.5 mM; SDS, 2%; protease inhibitor cocktail (Sigma #11873580001); phosphatase inhibitors (Sigma #4906845001)) using zirconium beads (Benchmark Scientific #D1132-30) in a bead homogenizer. Protein concentrations were measured using the BCA Protein Assay Kit (Thermo Fisher #23227). Whole cell extracts were separated by sodium dodecyl sulfate-polyacrylamide gel electrophoresis and transferred to polyvinylidene difluoride

membranes (Millipore #IPVH00010). The antibodies and dilutions used were as follows: Arginase-1 (1:1500, CST #93668), CD206 (1:1000, CST #24595T), FGFR1 (1:1500, CST #9740S), Sox4 (1:4000, Diagenode #C15310129), GAPDH (1:4000, CST #2118), and β-actin (1:4000, CST #3700S). GAPDH and β-actin served as loading controls.

Tandem mass tag mass spectrometry (TMT MS) and data processing

Sample preparation for deep scale proteomic profiling was performed as described previously by the CPTAC consortium (1) with slight variations. Briefly, frozen tumor tissues were crushed to powder and lysed with urea lysis buffer containing 8 M urea (G-Biosciences #BC89), 75 mM NaCl, 50 mM Tris pH 8.0, 1 mM EDTA (Sigma #E7889), 2 µg/ml Aprotinin (Sigma #A6103), 10 µg/ml Leupeptin (Roche #11017101001), 1 mM PMSF (Sigma #93482-50ML-F), 10 mM NaF, phosphatase inhibitor cocktail 2 (Sigma #P5726), and phosphatase inhibitor cocktail 3 (Sigma #P0044). Samples were lysed for 30 min on ice followed by 2 cycles of 5 sec ON/10 sec OFF sonication (Sonics Materials #GE 505). The lysates were cleared by centrifugation, and the protein concentration was measured using a NanoDrop spectrophotometer (DeNovix #DS-11). Total protein (150 µg) from each sample was reduced with 5 mM dithiothreitol (DTT) for 1 hr at 37°C and alkylated with 10 mM iodoacetamide (Sigma #I1149) for 45 min in the dark at RT. The samples were then diluted with 50 mM Tris pH 8.0, and subjected to Lysyl EndopeptidaseR (Wako #129-02541) digestion for 2 hrs and trypsin (Thermo Fisher #90057) digestion overnight at RT. The digest was acidified with 1% formic acid (Fisher #A117-50) and desalted using Sep-Pak Vac 1cc C18 cartridges (Waters #WAT054955). Elutes were dried with SpeedVac (Thermo Fisher #SC210A) and dissolved in 50 mM HEPES pH 8.5 buffer (Alfa Aesar #J63218).

For TMT labeling, 120 µg digested peptide from each sample as well as RefMix, which is a mixture of equal amounts of peptide from each sample, were labeled with the TMT10plex Label Reagent Set (Thermo Fisher #90110) for 1 hr at RT. After confirming the labeling efficiency for each channel using quality control MS runs, the reaction was quenched by adding 5% hydroxylamine (Thermo Fisher #90115) for 15 min at RT. All samples were then combined and freeze dried using SpeedVac. Each plex was reconstituted with 1 ml of 3% ACN/0.1% formic acid and desalted using Sep-Pak Vac 3cc tC18 cartridges (Waters #WAT054925). The elute

was dried using SpeedVac.

TMT-labeled peptides were fractioned offline using an Agilent 300Extend-C18 column (4.6 mm X 250 mm, 5 µm) coupled to an Agilent 1260 Infinity II system at 1 ml/min for 96 min. The 96 fractions were concatenated into 24 peptide pools and a flow-through pool and acidified with 0.1% formic acid. The peptides were separated on an online nanoflow Easy-nLC-1200 system (Thermo Fisher) coupled to an Orbitrap Fusion Lumos ETD mass spectrometer (Thermo Fisher). Proteome fractions (1 µg each) were loaded onto pre-column (2 cm x 100 µm I.D.) and separated on in-line 5 cm x 150 µm I.D. column (Reprosil-Pur Basic C18aq, Dr. Maisch) equilibrated with 0.1% formic acid. Peptide separation was performed at a flow rate of 750 nl/min over a 90 min gradient time with different concentrations of solvent B (4-32% for 85 min, followed by 5 min wash at 90% B). The peptides were ionized at a positive spray voltage of 2.4 kV and the ion transfer tube temperature was set at 300°C. The mass spectrometer was operated in data-dependent mode with 2 sec cycle time. MS1 was acquired in Orbitrap (60000 resolution, scan range 350-1800 m/z, AGC 5e5, 50 ms injection time), followed by MS2 in Orbitrap (50000 resolution, AGC 1e5, 105 ms injection time, HCD 38%). Dynamic exclusion was set to 20 sec and the isolation width was set to 0.7 m/z.

To process the proteomics data, raw files were converted to mzML using MSConvert (2). MASIC (3) was used to calculate precursor ion intensities (derived from the area under each elution curve) and to extract reporter ion intensities using default high resolution MS parameters. The Butterworth smoothing method was used with a sampling frequency of 0.25 and an SIC tolerance of 10 ppm. Reporter ion tolerance was set to 0.003 Da with reporter ion abundance correction enabled. Raw spectra were searched with MSFragger (v3.2) using both mass calibration and parameter optimization (4, 5). Peptide validation was performed using a semi-supervised learning procedure in Percolator (6) as implemented in MokaPot (7). The peptides were grouped and quantified into gene product groups using gpGrouper (8). Only the gene products identified in both TMT multiplexes were retained for downstream analyses. Samples were first normalized to the internal reference within each TMT multiplex and then normalized by their median peptide abundance before subsequent data analyses.

Gene set enrichment analysis (GSEA)

GSEA (v3.0) was performed using hallmark gene sets (v7.0) from the Molecular Signature Database (MSigDB) using default settings after mapping mouse genes to their human homologs using the HomoloGene system. Genes without mapping were excluded, and the median value was taken when multiple mouse genes mapped to a single human gene. Pathway enrichment *P* values were calculated using gene set permutation.

Quantitative real-time PCR (qPCR)

Total RNA was extracted using TRIzol reagent (Thermo Fisher #15596026) following the manufacturer's instructions. Total RNA (1 µg) was converted to cDNA using the High-Capacity cDNA Reverse Transcription Kit (Thermo Fisher #4368814). The mRNA levels were detected using amfisure qGreen Q-PCR Master Mix (GenDEPOT #Q5602). *18S* was used as the internal reference gene for patient biopsy samples and *Actb* was used as the internal reference for all other samples. The levels of target genes were normalized to those of internal reference gene to calculate the $2^{-\Delta\Delta Ct}$ value. The sequences of all the qPCR primers are listed in Supplementary Table S6.

RNA silencing assays

In siRNA knockdown assays, 50 µM siRNA (Sigma) was reverse transfected into cells for 48 hrs using RNAiMax (Thermo Fisher #13778030). The following siRNAs were used in this study: si-mmu-Sox4-1 (Sigma #SASI_Mm01_00114970), si-mmu-Sox4-2 (Sigma # SASI_Mm01_00114972), si-has-SOX4 (Sigma # SASI_Hs01_00188751) and siRNA Universal Negative Control #1 (Sigma # SIC001).

References

1. Mertins P, Tang LC, Krug K, Clark DJ, Gritsenko MA, Chen L, et al. Reproducible workflow for multiplexed deep-scale proteome and phosphoproteome analysis of tumor tissues by liquid chromatography-mass spectrometry. *Nat Protoc.* 2018;13(7):1632-61.

2. Chambers MC, Maclean B, Burke R, Amodei D, Ruderman DL, Neumann S, et al. A cross-platform toolkit for mass spectrometry and proteomics. *Nat Biotechnol.* 2012;30(10):918-20.

3. Monroe ME, Shaw JL, Daly DS, Adkins JN, and Smith RD. MASIC: a software program for fast quantitation and flexible visualization of chromatographic profiles from detected LC-MS(/MS) features. *Comput Biol Chem.* 2008;32(3):215-7.

4. Kong AT, Leprevost FV, Avtonomov DM, Mellacheruvu D, and Nesvizhskii AI. MSFragger: ultrafast and comprehensive peptide identification in mass spectrometry-based proteomics. *Nat Methods.* 2017;14(5):513-20.

5. Yu F, Teo GC, Kong AT, Haynes SE, Avtonomov DM, Geiszler DJ, et al. Identification of modified peptides using localization-aware open search. *Nat Commun.* 2020;11(1):4065.

6. Anderson DC, Li W, Payan DG, and Noble WS. A new algorithm for the evaluation of shotgun peptide sequencing in proteomics: support vector machine classification of peptide MS/MS spectra and SEQUEST scores. *J Proteome Res.* 2003;2(2):137-46.

7. Fondrie WE, and Noble WS. mokapot: Fast and Flexible Semisupervised Learning for Peptide Detection. *J Proteome Res.* 2021;20(4):1966-71.

8. Saltzman AB, Leng M, Bhatt B, Singh P, Chan DW, Dobrolecki L, et al. gpGrouper: A Peptide Grouping Algorithm for Gene-Centric Inference and Quantitation of Bottom-Up Proteomics Data. *Mol Cell Proteomics.* 2018;17(11):2270-83.

Supplementary figure legends

Supplementary Figure 1. Zotatifin inhibits tumor growth in a cohort of *Trp53*-null preclinical models. **A,** Individual tumor growth curves of BALB/c mice treated with either vehicle or Zotatifin. **B,** Body weight changes of tumor-bearing BALB/c mice over the treatment course. In **A** and **B**, n=6 biological replicates for 2225L-LM2 and n=5 for all other models in each treatment arm. **C,** Growth curves of 4T1 and E0771 tumors treated with either vehicle or Zotatifin. **D,** Body weight changes of 4T1 and E0771 tumor-bearing mice over the treatment course. In **C** and **D**, n=5 biological replicates for both models. In **A-D**, data are presented as mean ± SEM and analyzed using two-way ANOVA with Bonferroni's multiple comparison test. **E** and **F,** Left, representative images of IHC staining of BrdU in ethical endpoint 2225L-LM2 (**E**) or 2208L(**F**) tumor tissues. Scale bar, 50 µm. Right, quantification of IHC staining. Five representative 20X images were analyzed for each tumor. Data are presented as mean ± SEM and analyzed using two-tailed unpaired Student's *t*-test.

Supplementary Figure 2. Zotatifin alters the tumor immune microenvironment. **A** and **B,** Left, representative images of IHC staining of S100A8 in 2225L-LM2 (**A**) or 2208L (**B**) treated with vehicle or Zotatifin till ethical endpoint. Scale bar, 50 µm. Right, quantification of IHC staining. Three to six representative 20X images were analyzed for each tumor. N=5 biological replicates per group. Data are presented as mean ± SEM and analyzed using two-tailed unpaired Student's *t*-test. **C,** IHC staining of F4/80 in 2153L tumors treated with indicated therapy. Scale bar, 100 µm. **D,** Growth curves of 2153L tumors treated with indicated therapy. n=4 biological replicates for the Zotatifin+anti-Csf1r arm and n=3 for all other arms.

Supplementary Figure 3. Zotatifin inhibits the translation of *Sox4* and *Fgfr1* mRNAs. **A,** Immunoblotting analysis of 4T1 tumors that were treated with vehicle or Zotatifin in vivo. n = 5 biological replicates per group. **B,** Immunoblotting analysis of 2153L and E0771 cells that were treated with 40 nM Zotatifin in vitro for 1 hr or 6 hrs respectively. **C** and **D,** QPCR analysis for *Sox4* (**C**) and *Fgfr1* (**D**) in 2153L cells that were treated with different concentrations of Zotatifin for 6 hrs in vitro. **E,** Immunoblotting analysis of 2153L cells that were

treated with CHX or MG132 for different time periods. **F** and **G,** QPCR analysis for *SOX4* (**F**) and *FGFR1* (**G**) in BT549 cells that were treated with 40 nM Zotatifin for different time periods. **H,** Immunoblotting analysis of 2153L cells that were treated with indicated drugs for 24 hrs. CPT, camptothecin. **I,** QPCR analysis of HAP1 cells that were treated with 40 nM Zotatifin for 6 hrs. In **C**, **D**, **F**, **G**, and **I**, data are representative of two independent experiments and are presented as mean ± SD of technical triplicates. **J,** Distribution of *Actb* and *Gapdh* mRNAs across the different fractions in polysome profiling analysis of 2153L cells that were treated with vehicle or 40 nM Zotatifin for 2 hrs. **D**ata are presented as mean ± SEM of three biological replicates. **K,** Immunoblotting analysis of 2153L cells that were treated with vehicle or Zotatifin in the presence of CHX or MG132 for 2 hrs. Data are representative of two independent experiments.

Supplementary Figure 4. Zotatifin induces interferon response genes. **A,** QPCR analysis of 2153L cells that were treated with different concentrations of Zotatifin for 6 hrs. **B,** QPCR analysis of 2153L cells that were treated with 40 nM Zotatifin for different time periods. In **A** and **B**, data are representative of two independent experiments and are presented as mean ± SD of technical triplicates. **C,** QPCR analysis of 2153L cells that were treated with indicated drugs for 24 hrs. Data are presented as mean ± SD of technical triplicates. **D,** QPCR analysis of paired ER+ breast cancer biopsies from pre-treatment (pre) and on Zotatifin treatment (on) patients. The mRNA levels of pre-treatment samples were set as 1 and fold changes were calculated for each paired sample. Data are analyzed using two-tailed unpaired Student's *t*-test. n=8 patient biopsy pairs.

Supplementary Figure 5. Sox4 inhibition by Zotatifin contributes to Zotatifin induced interferon response genes. **A,** The mRNA levels of *Eif4a1*, *Eif4a2,* and *Ddx3x* in normal mammary glands of BALB/c mice and *Trp53*-null preclinical models. The RNA levels for each gene were averaged RNA-seq signals from 1-12 biological replicates. **B,** The Chronos dependency scores of *EIF4A1* and *EIF4A2* in CRISPR knockout screens. A lower Chronos score indicates higher essentiality. A score of 0 indicates a gene is not essential and a score of -1 is the median scores of all pan-essential genes. **C,** The RNA and protein levels of *EIF4A1*, *EIF4A2*, and

DDX3X in non-triple-negative or triple-negative breast cancer tissues from the Clinical Proteomic Tumor Analysis Consortium (CPTAC) database. **D,** Immunoblotting analysis of 2153L cells that were transfected with siRNA for 48 hrs and left untreated or treated with 40 nM Zotatifin for the last 24 hrs. **E,** Immunoblotting analysis of BT549 cells that were transfected with siRNA for 48 hrs. * denotes a non-specific band. **F,** QPCR analysis of BT549 cells that were transfected with negative control siRNA with or without Zotatifin treatment, or *SOX4* siRNAs without Zotatifin treatment for 48 hrs. **G,** QPCR analysis of Zotatifin-induced gene fold changes in BT549 cells that were transfected with negative control siRNA or *SOX4* siRNA in the presence of vehicle or Zotatifin. In **D-G**, data are representative of two independent experiments. In **F** and **G**, data are presented as mean ± SD of technical triplicates.

Supplementary Figure 6. Zotatifin synergizes with chemotherapy to suppress tumor progression. **A,** Top, treatment scheme of BALB/c mice. Bottom, individual growth curves of 2153L tumors treated with indicated drugs. Each line represents a tumor and different line colors denote separate experimental batches. Data from 3 to 5 independent experimental batches are integrated. n=24 for Vehicle, n=22 for Zotatifin, n=13 for Carboplatin, and n=24 for Zotatifin+Carboplatin. **B,** Body weight changes of 2153L tumor-bearing BALB/c mice over the treatment course. n≥6 biological replicates per treatment arm. **C** and **D,** Left, representative images of IHC staining of BrdU (**C**) or cleaved Caspase 3 (**D**) in 2153L tumors that were treated with indicated drugs for 3 days. The regions outlined in box are magnified below. Scale bar, 50 µm. Right, quantification of IHC staining. Five representative 20X images were analyzed for each tumor. n = 3 biological replicates per group. Data are presented as mean ± SEM and analyzed using two-tailed unpaired Student's *t*-test. **E,** Tumor growth curves of 2153L tumors treated with indicated drugs. n≥4 biological replicates per treatment arm. **F,** Tumor growth curves of 2153L tumors treated with indicated drugs. n=5 biological replicates per treatment arm. In **B**, **E**, and **F**, data are presented as mean ± SEM and analyzed using two-way ANOVA with Bonferroni's multiple comparison test. **G,** Kaplan-Meier survival curves of 2153L tumor-bearing mice treated with indicated drugs. n=5 biological replicates per group. The log-rank test (two-tailed) was used to test for the significant differences

of curves between groups.

Supplementary Figure 7. Zotatifin and carboplatin combination therapy induces interferon response genes and changes in the tumor microenvironment. **A,** QPCR analysis of 2153L cells that were treated with 40 nM Zotatifin or/ and 10 □M carboplatin for 24 hrs in vitro. **B,** UMAP plot overlaid with the expression of selected markers from mass cytometry analysis of tumor-infiltrating immune cells from all treatment groups in 2153L.

Supplementary Figure 8. Zotatifin and carboplatin combination therapy induces T cell infiltration to the tumor microenvironment. **A,** Representative images of IHC staining of CD4 (top) and CD8 (bottom) in 2153L tumors from BALB/c mice that were treated with indicated drugs for 11 days. Scale bar, 50 µm. **B,** Quantification of CD4 (left) and CD8 (right) IHC staining. Five to eleven representative 20X images were analyzed for each tumor. n = 3 biological replicates per group. Data are presented as mean ± SEM and analyzed using two-tailed unpaired Student’s *t*-test.

Supplementary table legends

Supplementary Table S1. The proteomic alterations determined by tandem mass tag mass spectrometry in 2153L tumors treated with Zotatifin for 3 days compared with the vehicle. n=4 biological replicates per arm. Statistical significance was determined using a two-tailed unpaired moderated *t*-test.

Supplementary Table S2. Gene set enrichment analysis (GSEA) of pathways that are enriched in 2153L tumors treated with vehicle compared to Zotatifin using data from mass spectrometry. GSEA was performed with the MSigDB hallmarks dataset.

Supplementary Table S3. Gene set enrichment analysis (GSEA) of pathways that are enriched in 2153L tumors treated with Zotatifin compared to vehicle using data from mass spectrometry. GSEA was performed with the MSigDB hallmarks dataset.

Supplementary Table S4. Gene set enrichment analysis (GSEA) of pathways that are enriched in 2153L tumors treated with Zotatifin+carboplatin compared to Zotatifin monotherapy using data from mass spectrometry. GSEA was performed with the MSigDB hallmarks dataset.

Supplementary Table S5. Gene set enrichment analysis (GSEA) of pathways that are enriched in 2153L tumors treated with Zotatifin+carboplatin compared to carboplatin monotherapy using data from mass spectrometry. GSEA was performed with the MSigDB hallmarks dataset.

Supplementary Table S6. Sequences of qPCR primers.

Supplementary Figure 1

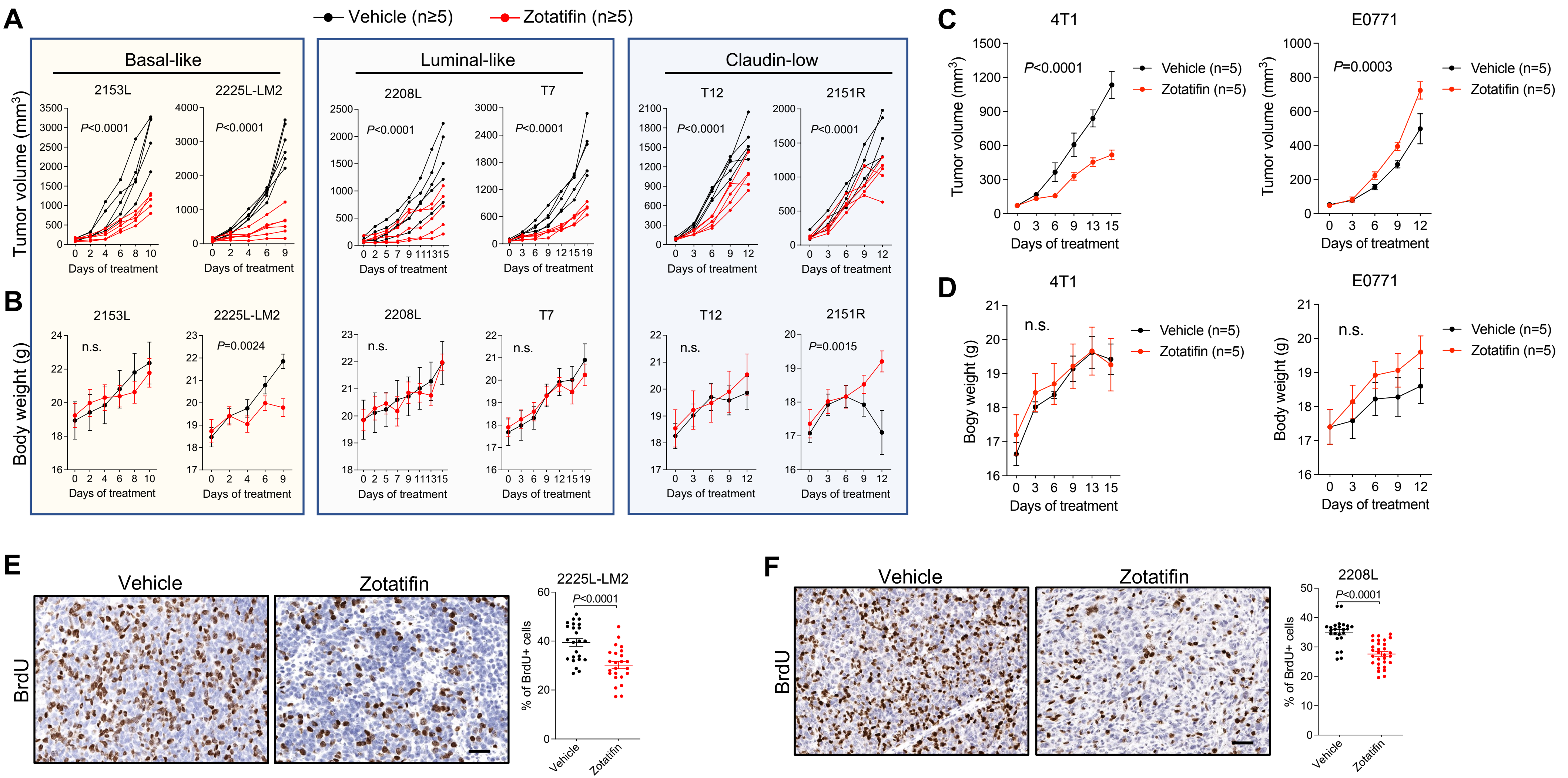

Supplementary Figure 2

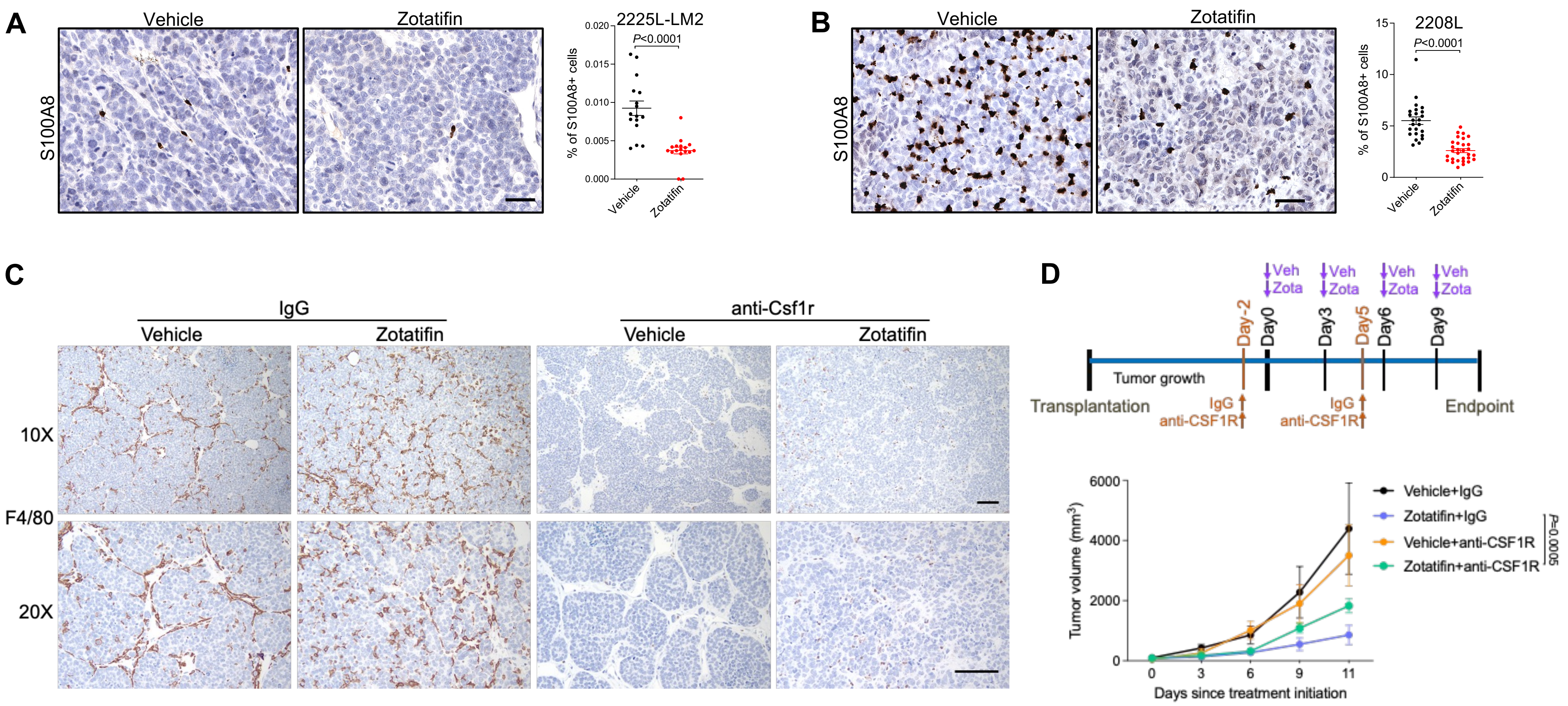

Supplementary Figure 3

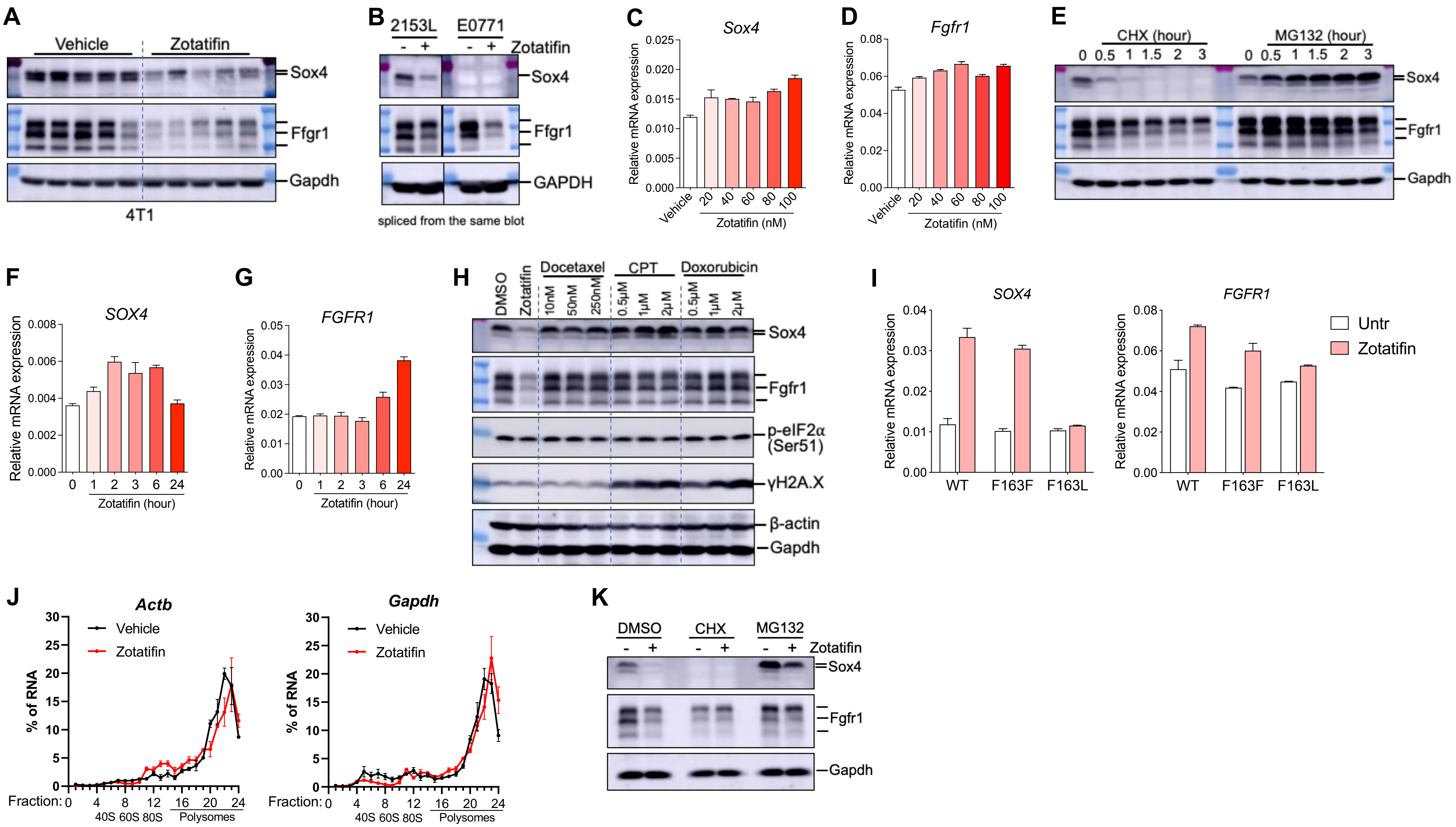

Supplementary Figure 4

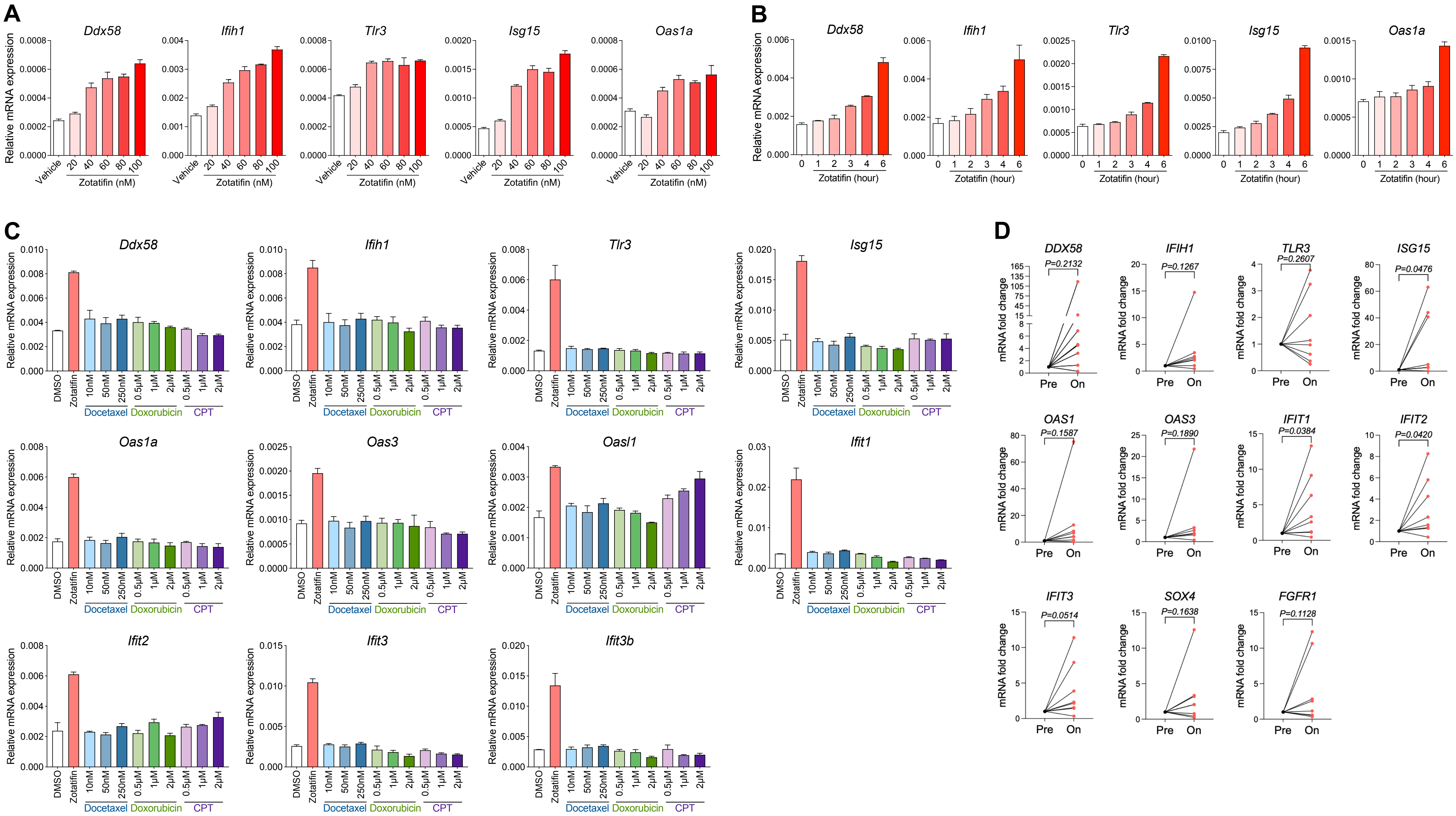

Supplementary Figure 5

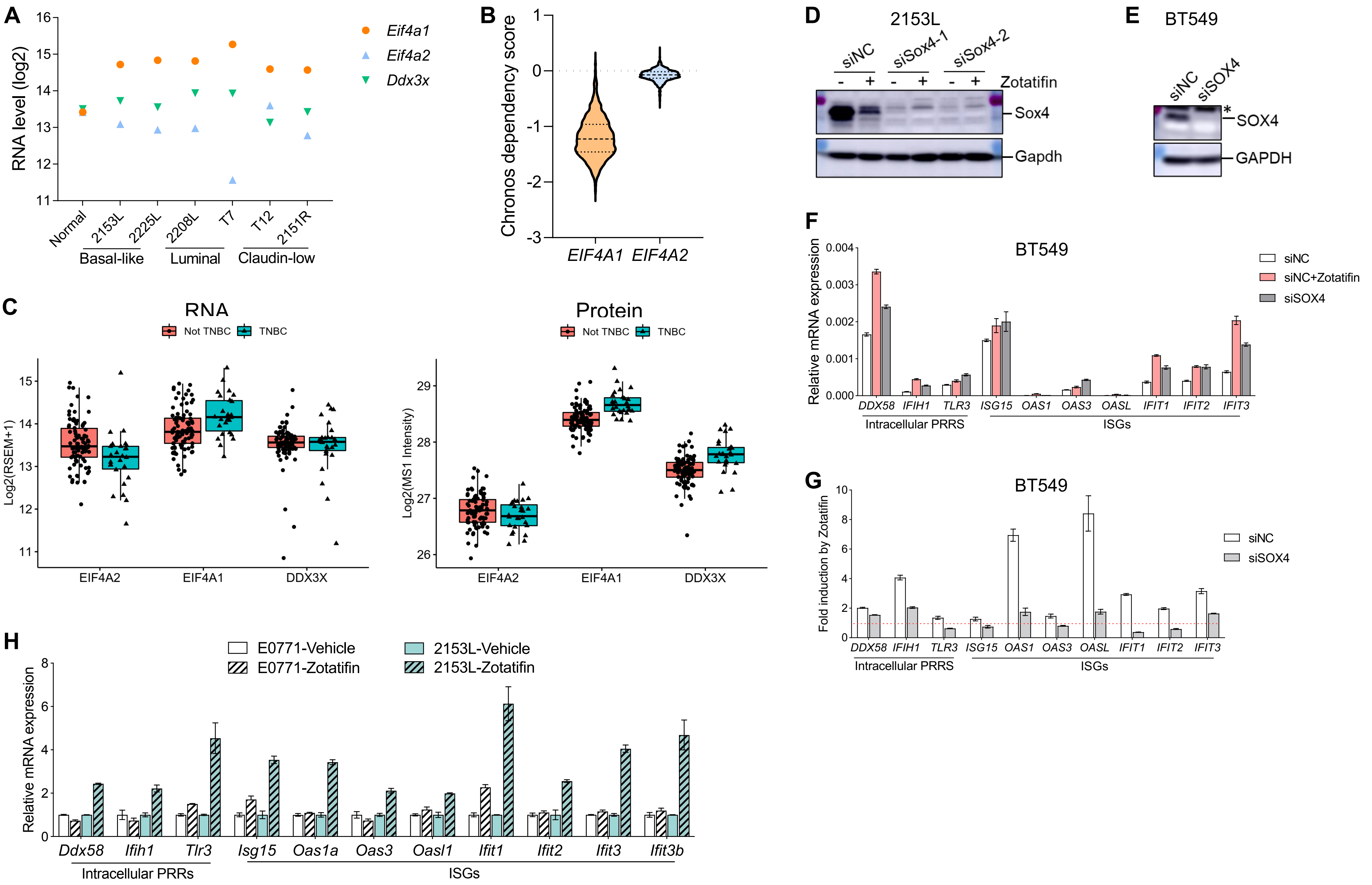

Supplementary Figure 6

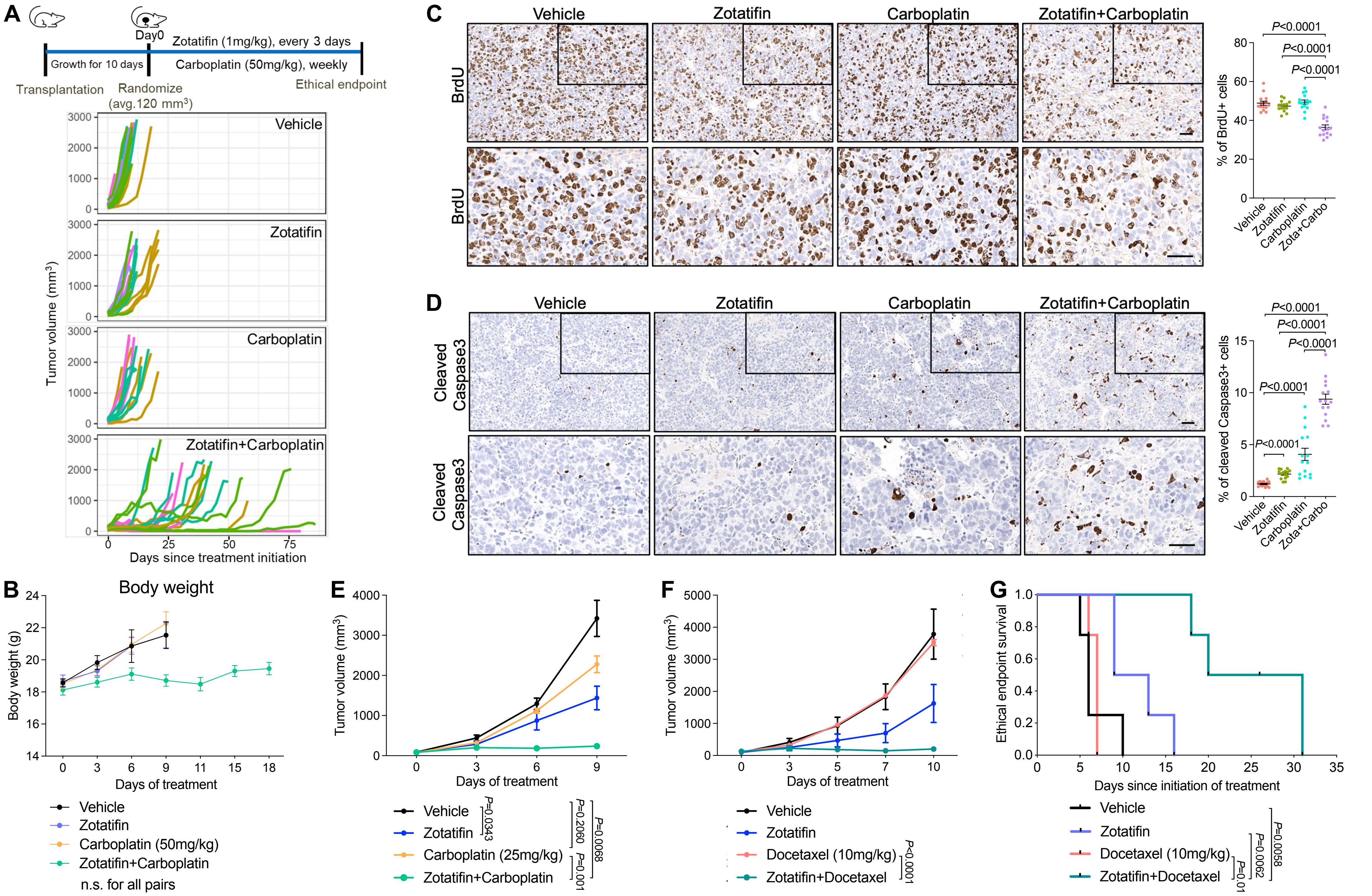

Supplementary Figure 7

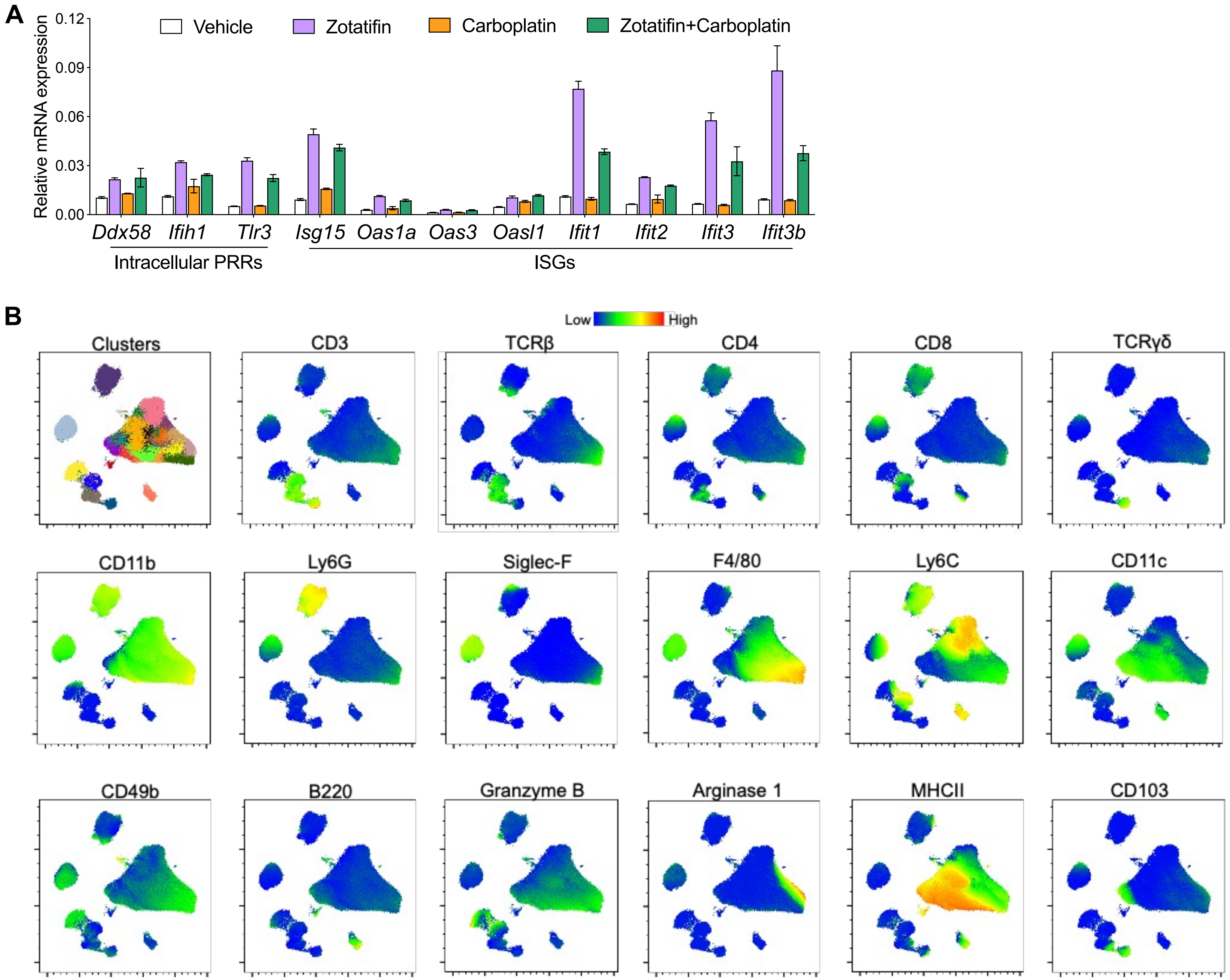

Supplementary Figure 8

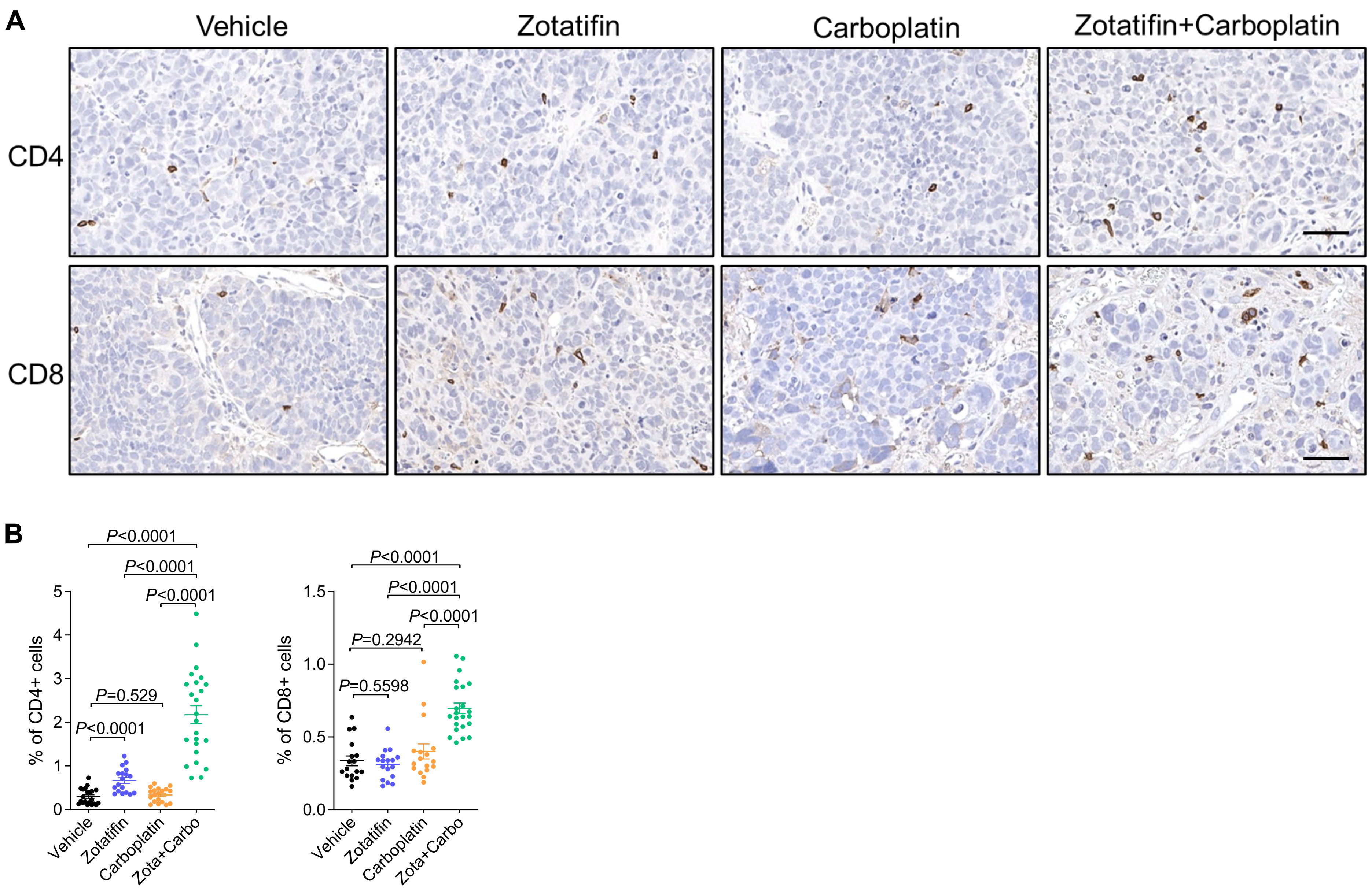

Figure 2I

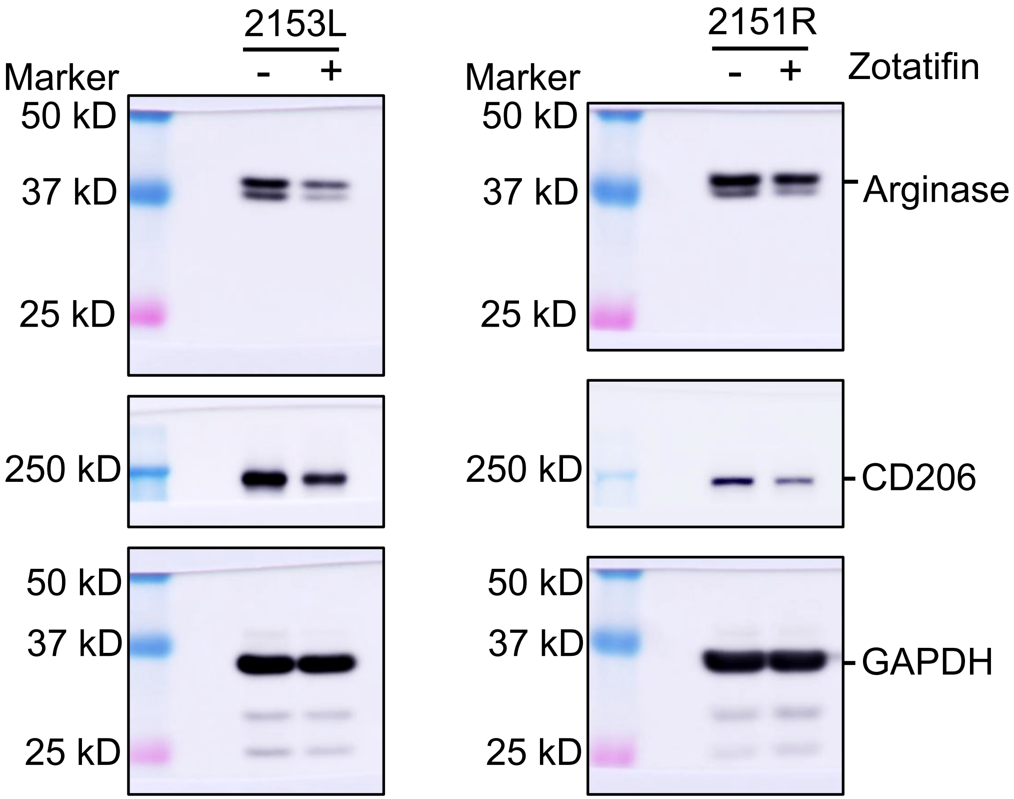

2153L
2151R
Marker
Marker
- +
- +
Zotatifin
50 kD
37 kD
25 kD
250 kD
50 kD
37 kD
25 kD
50 kD
37 kD
25 kD
250 kD
50 kD
37 kD
25 kD
Arginase
CD206
GAPDH

Figure 4A

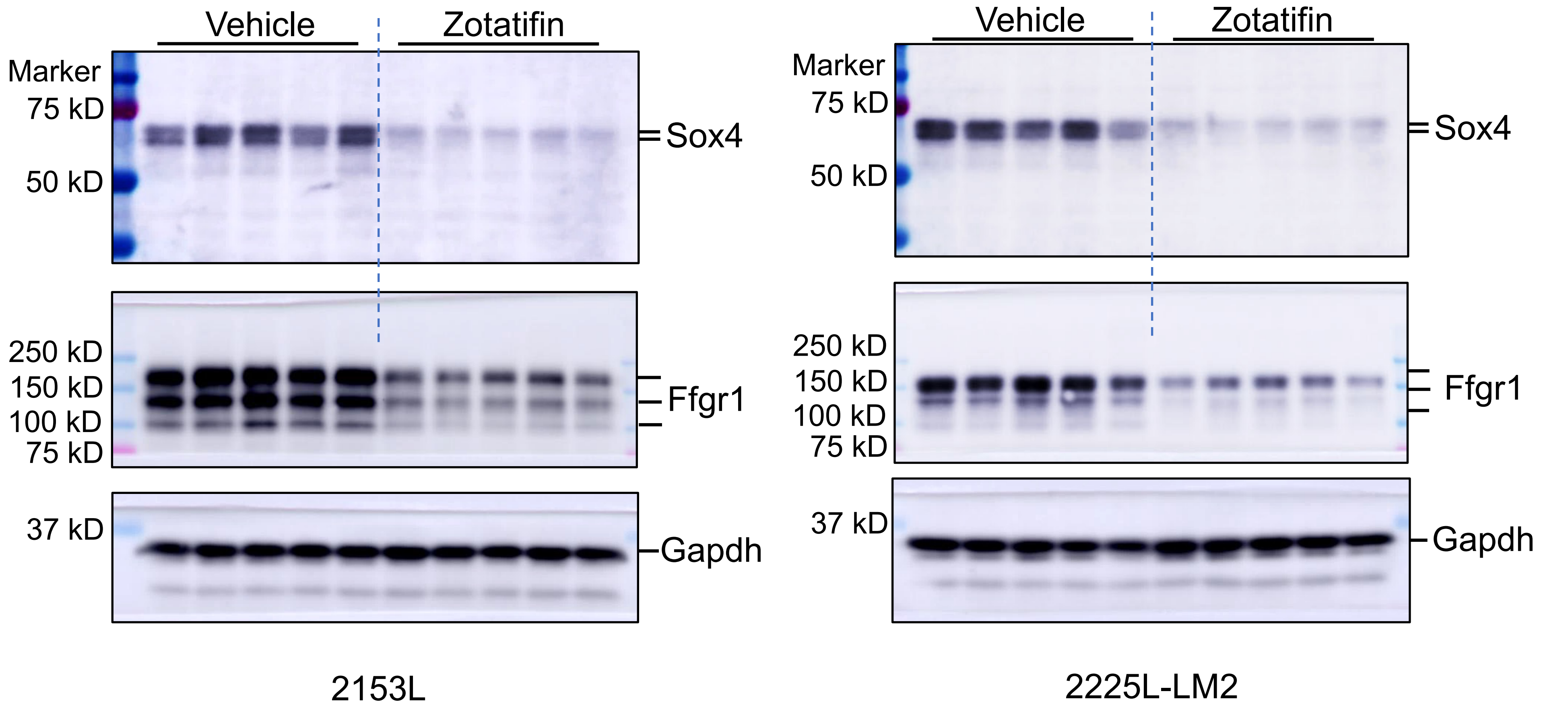
Vehicle
Zotatifin
Marker
75 kD
50 kD
Sox4
250 kD
150 kD
100 kD
75 kD
Ffgr1
37 kD
Gapdh
2153L
Vehicle
Zotatifin
Marker
75 kD
50 kD
Sox4
250 kD
150 kD
100 kD
75 kD
Ffgr1
37 kD
Gapdh
2225L-LM2

Figure 4A

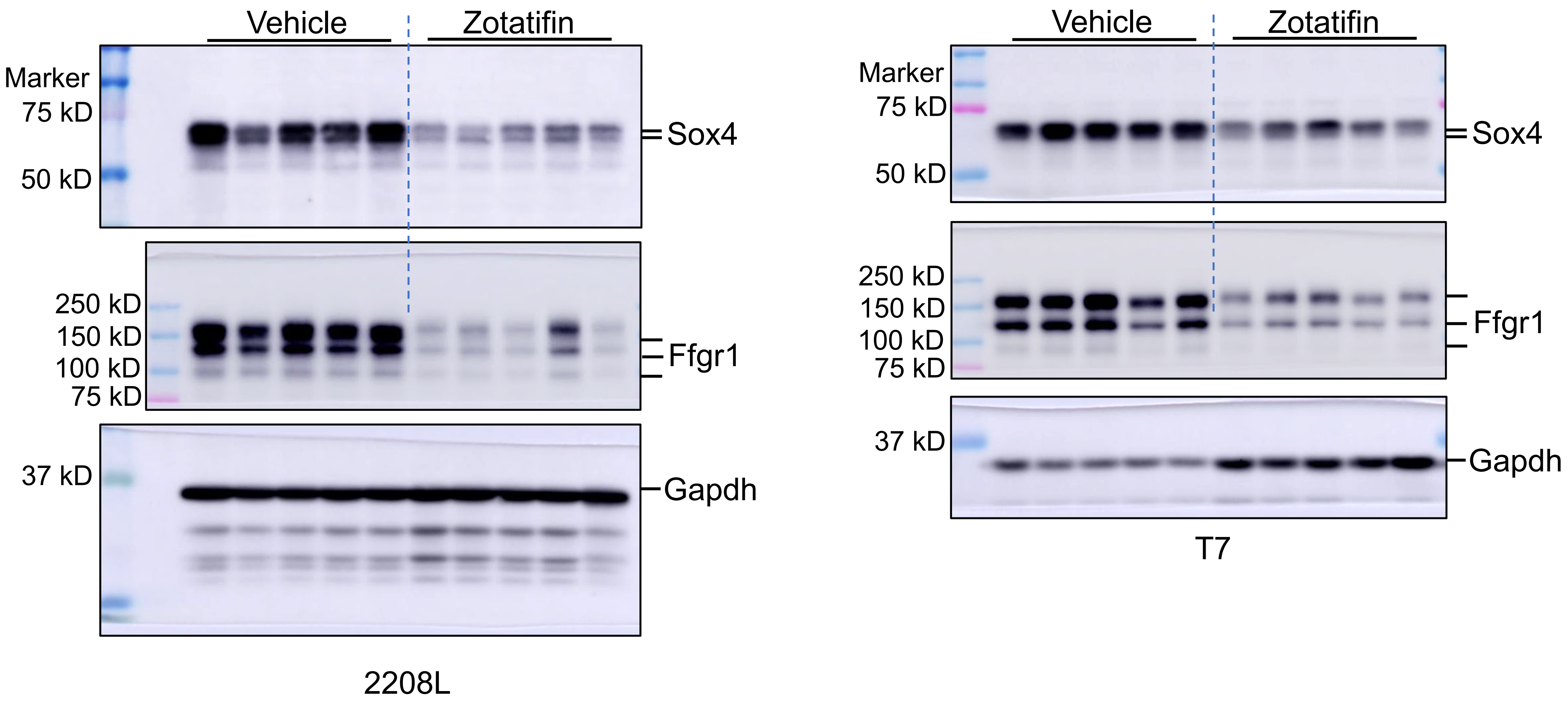
Vehicle
Zotatifin
Marker
75 kD
50 kD
Sox4
250 kD
150 kD
100 kD
75 kD
Ffgr1
37 kD
Gapdh
2208L
Vehicle
Zotatifin
Marker
75 kD
50 kD
Sox4
250 kD
150 kD
100 kD
75 kD
Ffgr1
37 kD
Gapdh
T7

Figure 4A

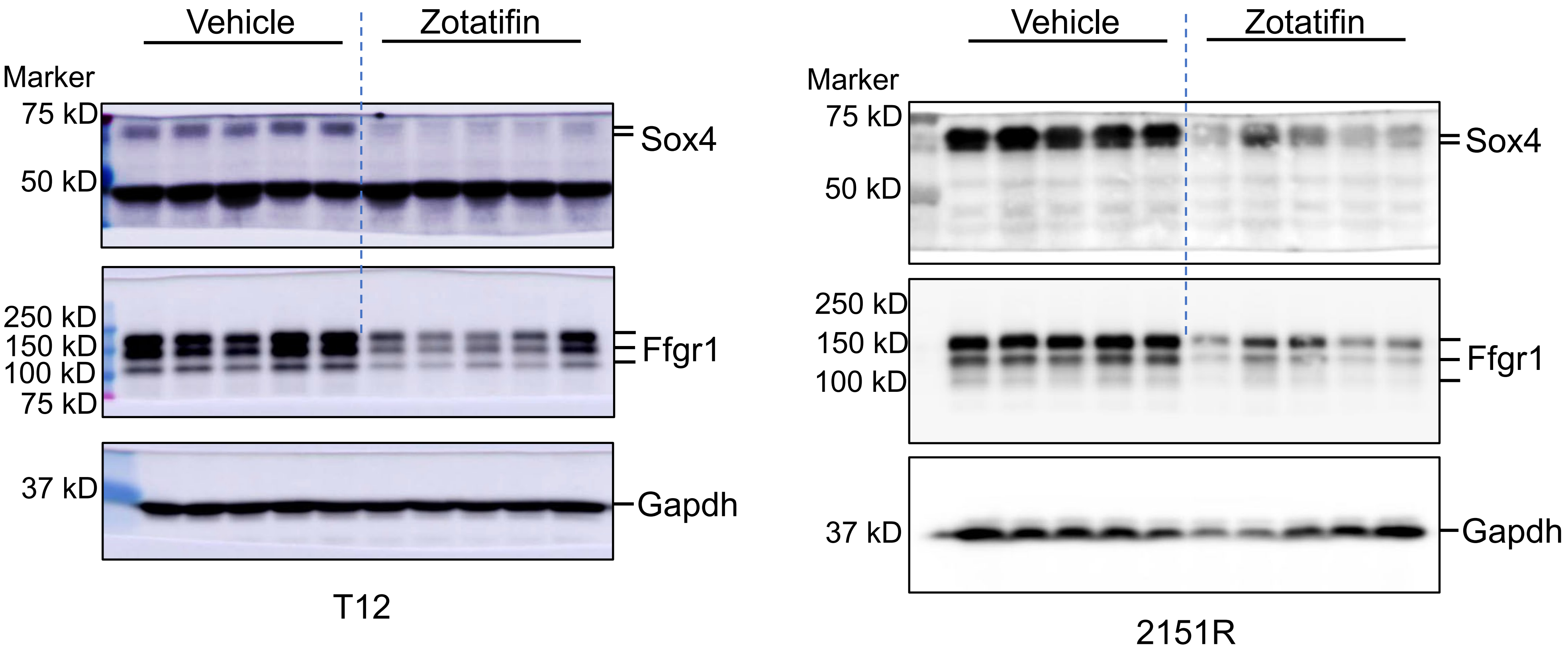

Vehicle
Zotatifin
Marker
75 kD
50 kD
Sox4
250 kD
150 kD
100 kD
75 kD
Ffgr1
37 kD
Gapdh
T12
Vehicle
Zotatifin
Marker
75 kD
50 kD
Sox4
250 kD
150 kD
100 kD
Ffgr1
37 kD
Gapdh
2151R

Figure 4D

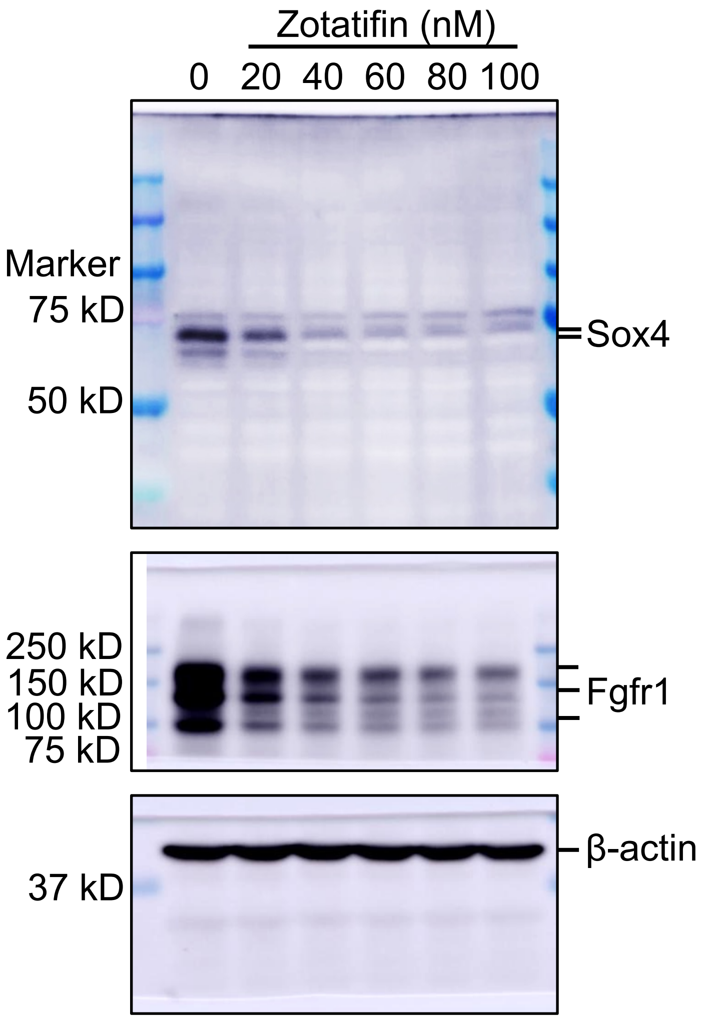
Zotatifin (nM)
0
20
40
60
80
100
Marker
75 kD
50 kD
Sox4
250 kD
150 kD
100 kD
75 kD
Fgfr1
β-actin
37 kD

Figure 4E

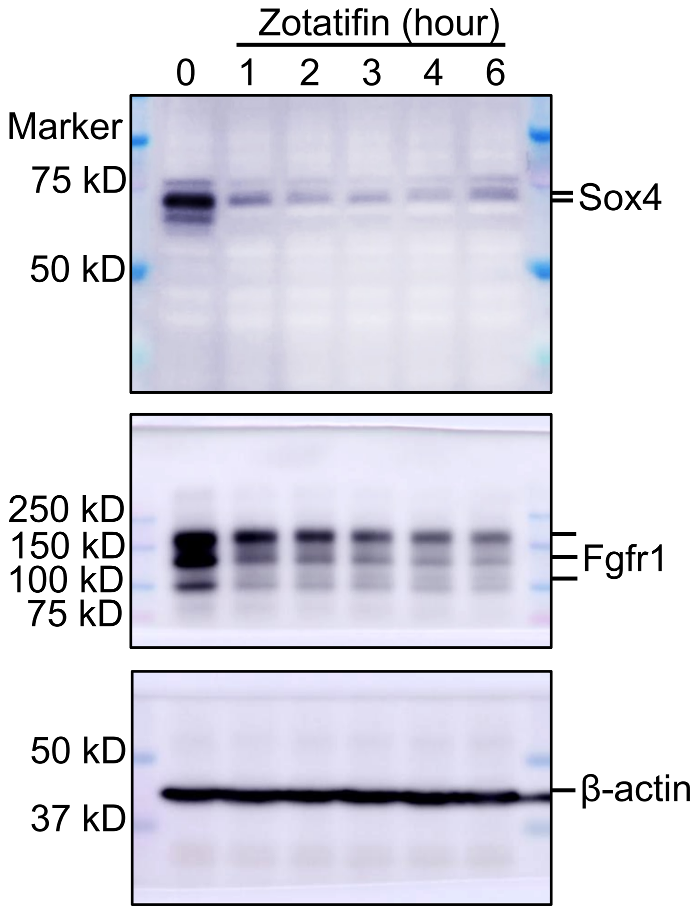
Zotatifin (hour)
0
1
2
3
4
6
Marker
75 kD
50 kD
Sox4
250 kD
150 kD
100 kD
75 kD
Fgfr1
50 kD
37 kD
β-actin

Figure 4F

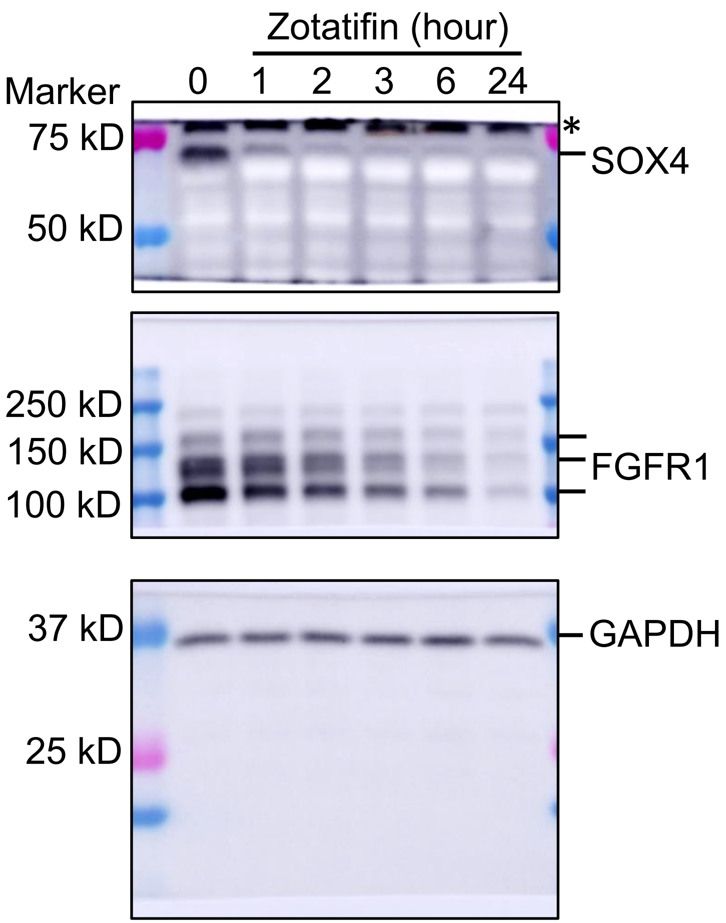

Zotatifin (hour)
Marker
0
1
2
3
6
24
75 kD
50 kD
*
SOX4
250 kD
150 kD
100 kD
FGFR1
37 kD
25 kD
GAPDH

Figure 4G

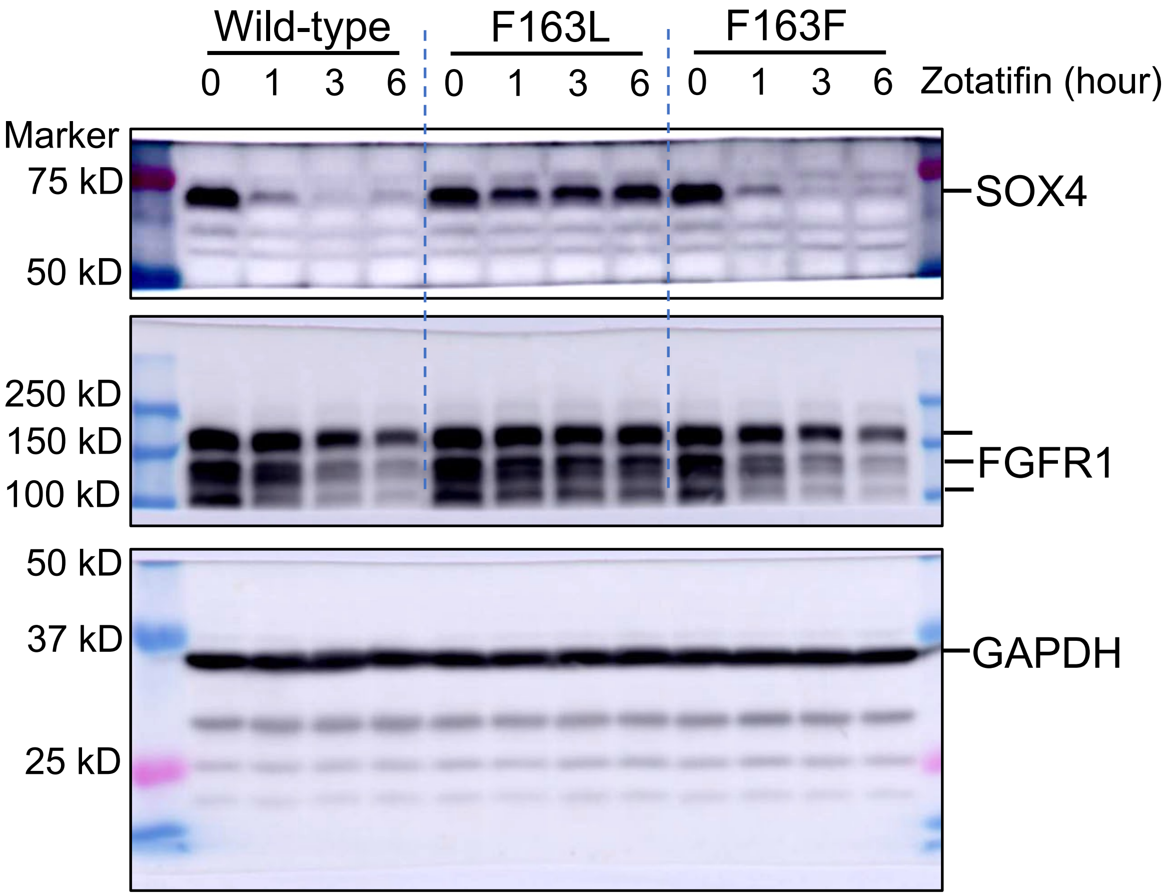
Wild-type
F163L
F163F
0 1 3 6 0 1 3 6 0 1 3 6 Zotatifin (hour)
Marker
75 kD
50 kD
SOX4
250 kD
150 kD
100 kD
FGFR1
50 kD
37 kD
25 kD
GAPDH

Supplementary Fig. S3A

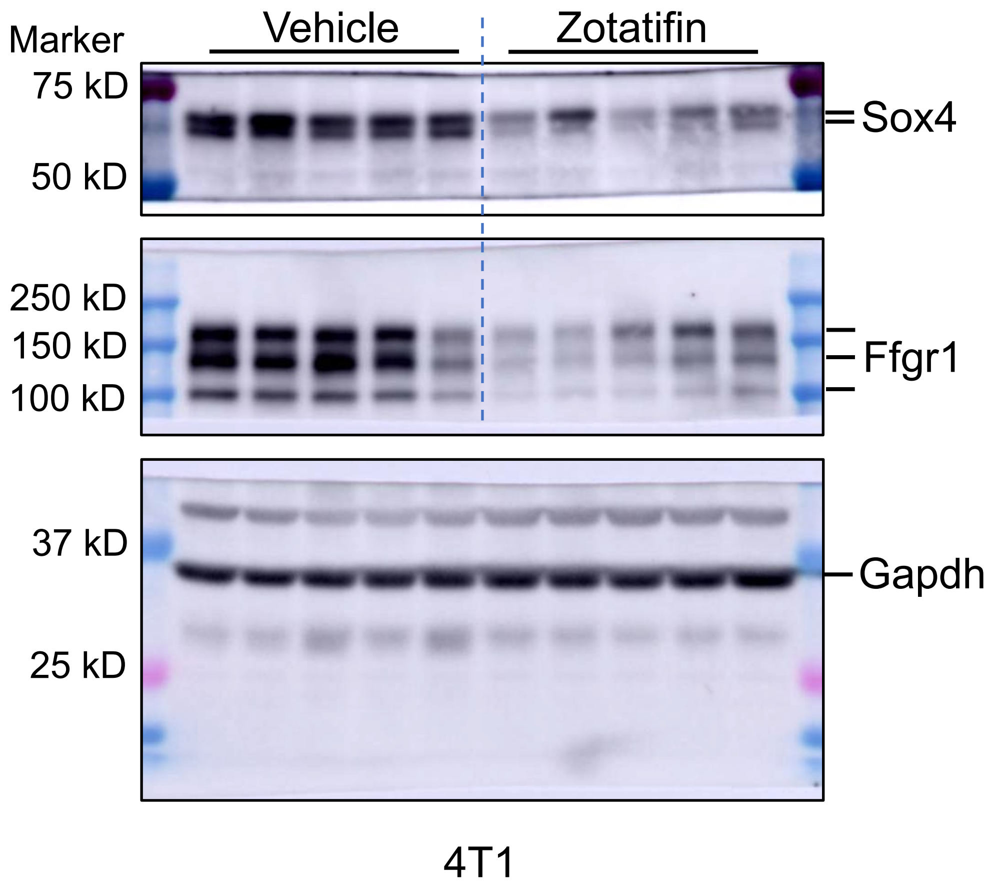

Supplementary Fig. S3B

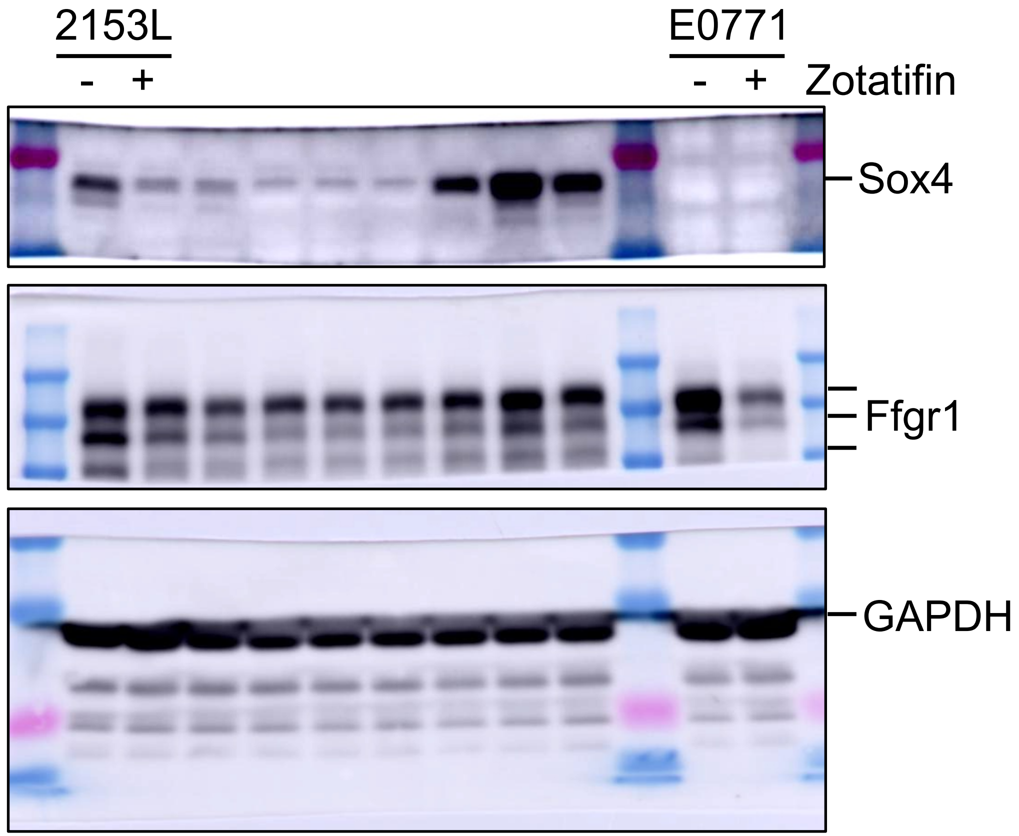

Supplementary Fig. S3E

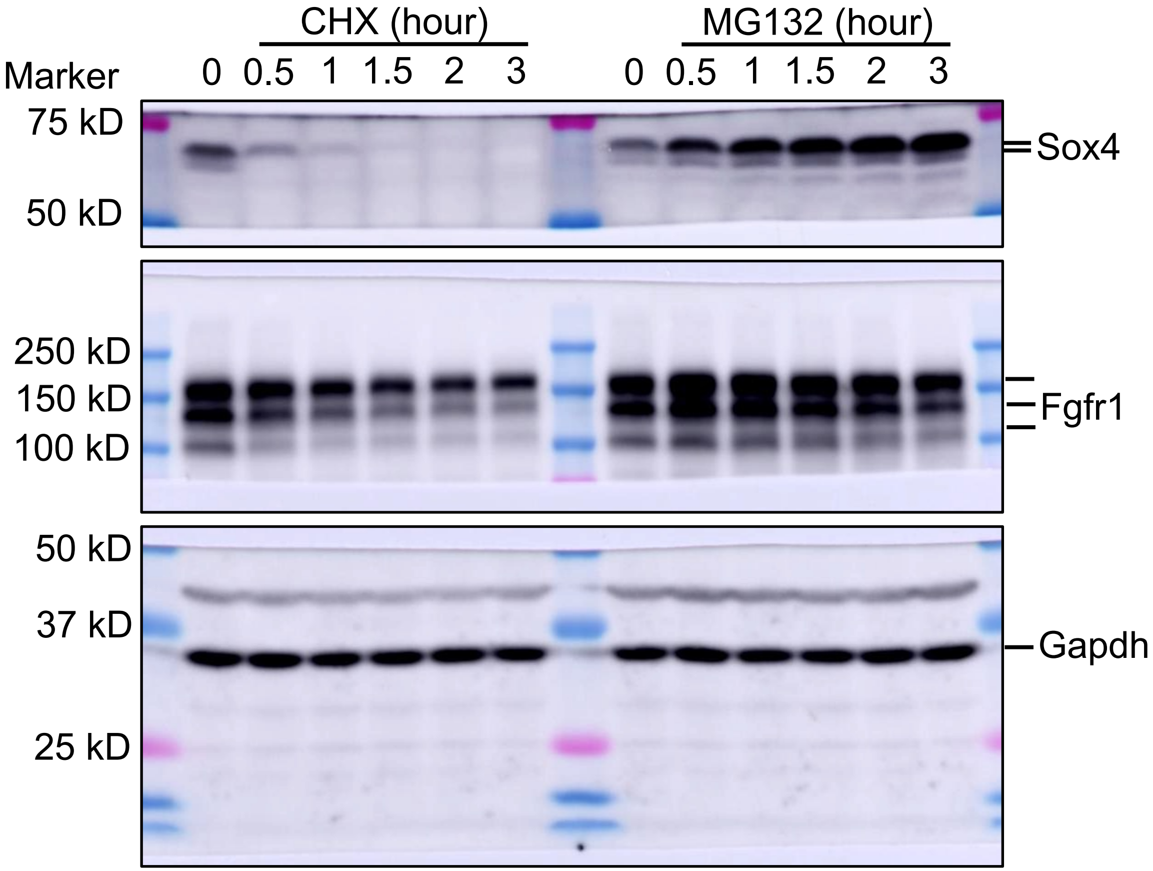

Supplementary Fig. S3H

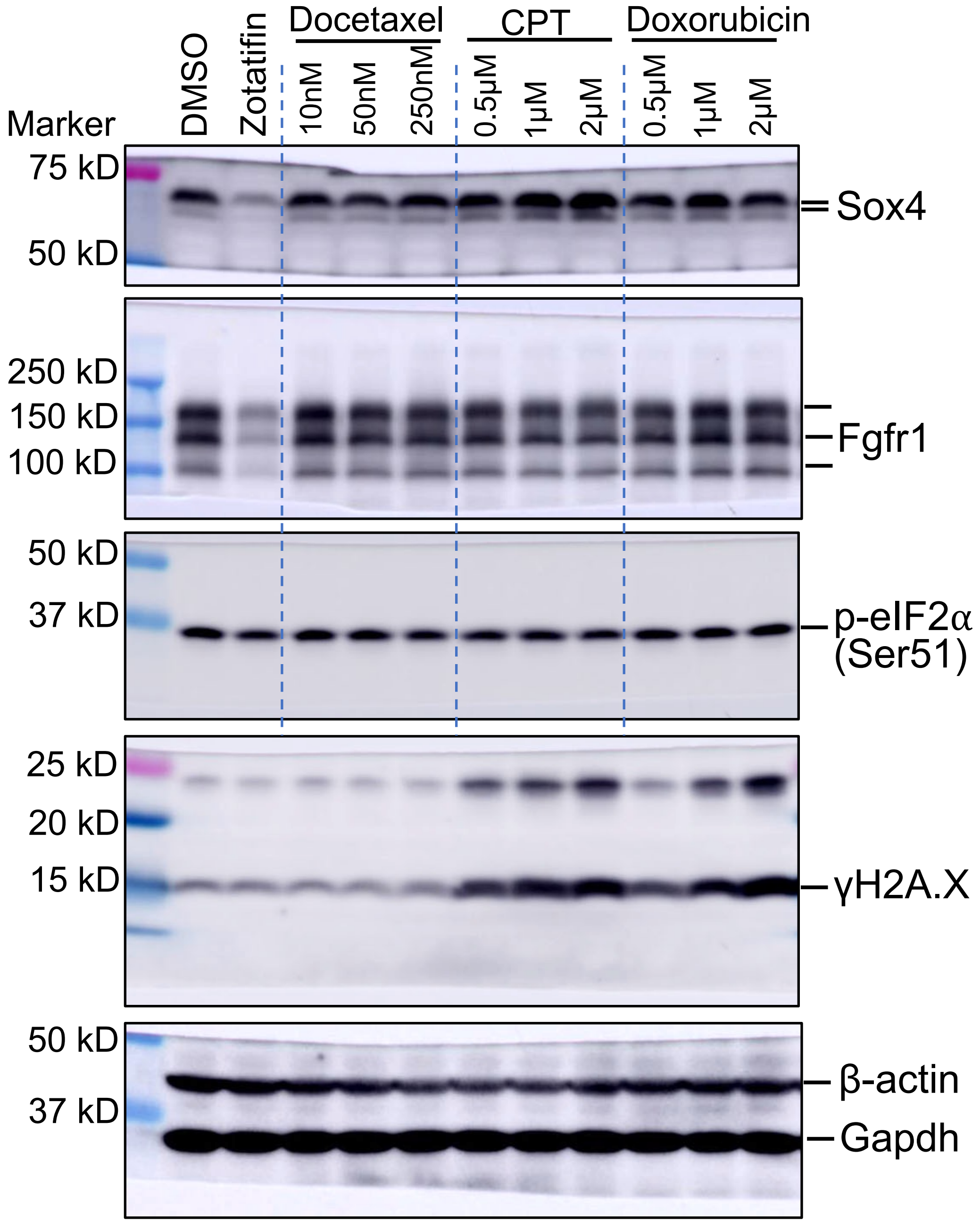

Supplementary Fig. S3K

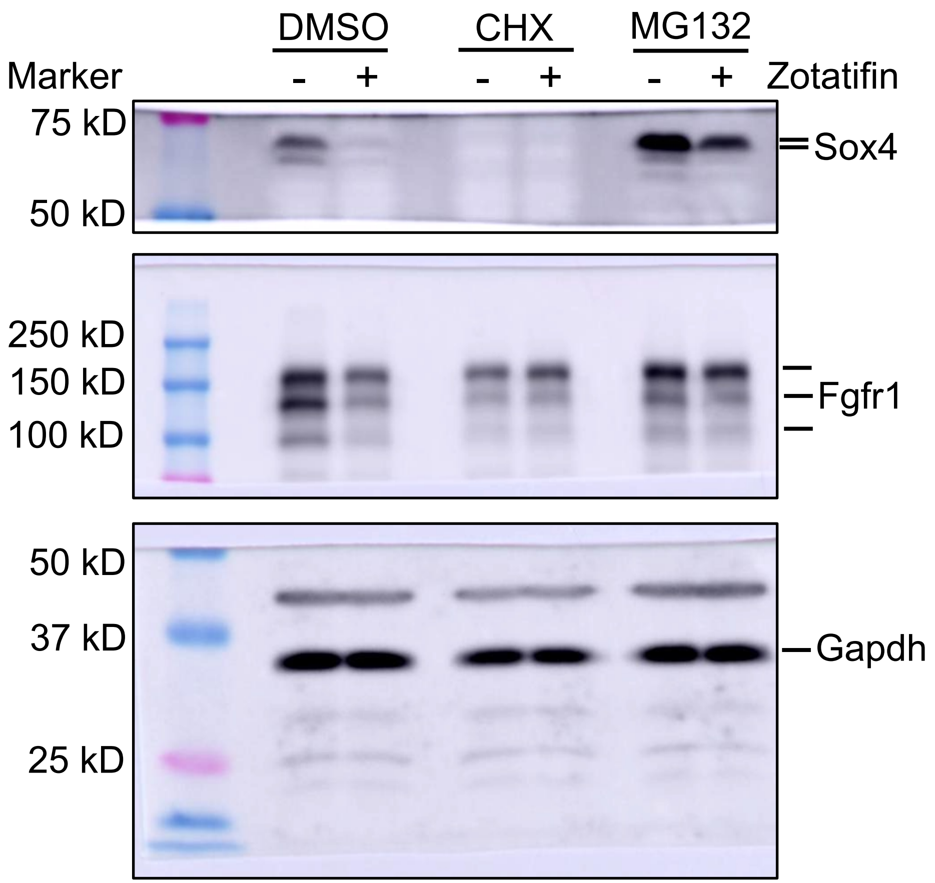

Supplementary Fig. S5D

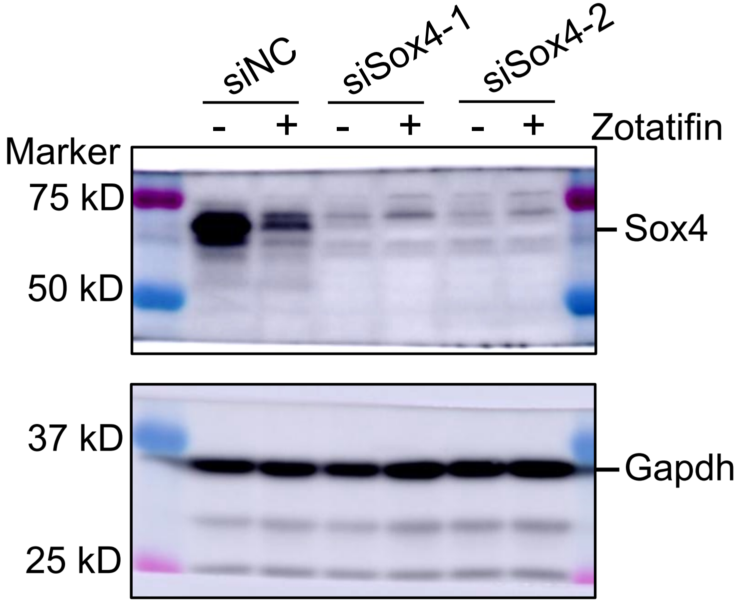

Supplementary Fig. S5E

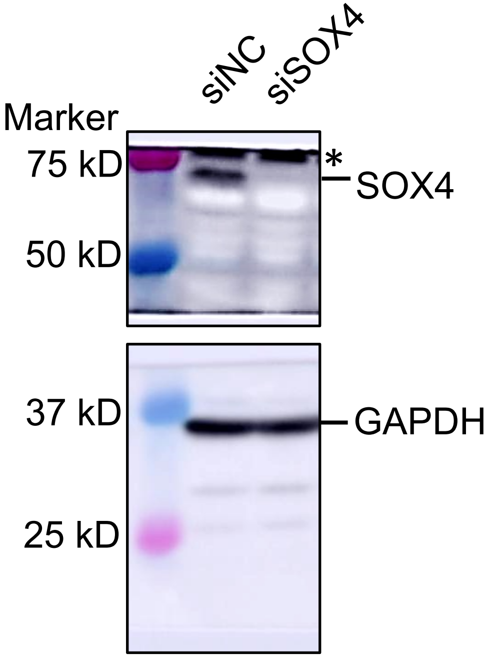

Supplement: Supplemental data [file jci-133-172503-s020.pdf]
